# Mapping vascular plasticity during fibrogenesis identifies fibrosis-associated endothelial cells in early-stage liver disease

**DOI:** 10.1172/jci.insight.207327

**Published:** 2026-09-01

**Authors:** Christina Gkantsinikoudi, Joshua P. Dignam, Raju Kumar, Elliot Jokl, Meenakshi Rana, Wenhao Li, Maryna Samus, Stephanie Landi, Varinder S. Athwal, Timothy J. Kendall, Antal Rot, Jonathan A. Fallowfield, Karen Piper Hanley, William Alazawi, Neil P. Dufton

**Affiliations:** 1 William Harvey Research Institute and; 2 Blizard Institute, Queen Mary University London, London, United Kingdom.; 3 University of Manchester, Manchester, United Kingdom.; 4 Manchester University NHS Foundation Trust, Manchester, England, United Kingdom.; 5 Centre for Inflammation Research, Institute for Regeneration & Repair, University of Edinburgh, Edinburgh, United Kingdom.

**Keywords:** Hepatology, Vascular biology, Endothelial cells, Fibrosis

## Abstract

Vascular plasticity is a crucial biological asset enabling our bodies to rapidly adapt to infections and acute inflammation. However, repeated insults during chronic disease can result in these vascular adaptations becoming irreversible, thereby driving disease progression and fibrosis. This study aimed to understand if phenotypic changes in endothelial cell (EC) identity could be indicative of progressive fibrosis and thereby offer diagnostic and therapeutic opportunities for patients with metabolic dysfunction–associated steatotic liver disease (MASLD). We integrated high-resolution imaging and proteomic and transcriptomic analysis, which collectively highlighted a central role for endothelial-mesenchymal transition–induced (EndMT-induced) EC plasticity in the derivation of fibrosis-associated EC (FAEC). We demonstrated that: (a) full-spectrum flow cytometry can provide new opportunities to categorize and phenotype EC subpopulations; (b) 2 distinct EndMT-derived FAEC subpopulations expanded during fibrogenesis (THY1.2^+^ICAM1^+^ and TAGLN^+^MCAM^+^ EC) that displayed unique immunomodulatory and metabolic phenotypes; (c) TAGLN^+^ FAEC are a conserved, profibrotic cell type that arose at early stages of MASLD; and (d) increased hepatic expression of TAGLN was significantly associated with detrimental patient outcomes at all stages of liver disease. This study paves the way for the development of FAEC-specific diagnostic and therapeutic approaches to tackle progressive fibrotic disease.

## Abstract 2

We describe how blood vessels change during fibrosis and identify markers that could help predict liver disease at earlier stages than current clinical tests.

## Graphical abstract

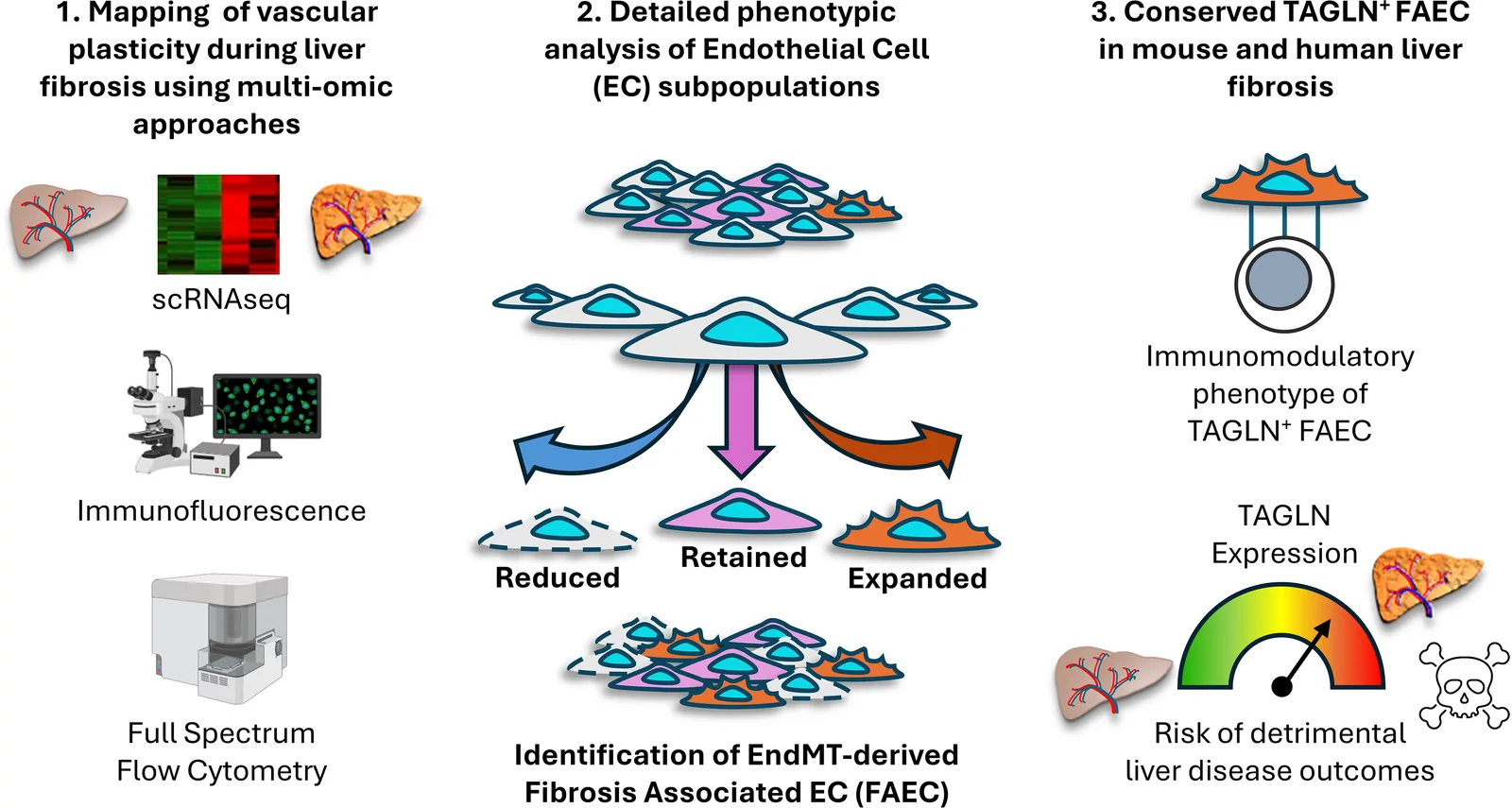

## Introduction

Fibrosis is the principal predictor of major adverse liver outcomes and of mortality in metabolic dysfunction–associated steatotic liver disease (MASLD). MASLD is the most common cause of chronic liver disease worldwide, and its prevalence has risen in parallel with rising levels of obesity and type II diabetes (1). Despite major advances, including 2 drugs licensed in the United States (2, 3), the 2 main unmet clinical challenges in MASLD are sensitive and accurate diagnostics and treatments with clinically significant effect sizes, both of which rely on improved understanding of pathology, including fibrogenesis.

Zonation of liver lobules containing specialized subpopulations of hepatic endothelial cells (EC), hepatocytes, and immune cells is critical for vascular, metabolic and immune homeostasis in the liver (4–6). Vascular zonation is dramatically impacted by chronic disease, with several studies documenting the changing EC landscape and the derivation of distinct endothelial cell clusters between healthy and fibrotic liver tissue in mice (7, 8) and patients (9, 10). Endothelial-mesenchymal transition (EndMT) is a central endothelial plasticity pathway responsible for phenotypic vascular changes (11–13). It is defined as the process by which EC lose expression of endothelial lineage markers, such as CD31, and upregulate mesenchymal markers such as transgelin (TAGLN) (14), THY1.2 (15) (also termed CD90.2), or platelet-derived growth factor receptor α (PDGFRα) (16). This process is predominantly driven through canonical TGF-β signaling and induces phenotypic changes such as enhanced expression of cytoskeletal and matrix-associated factors, including vimentin and collagen I and III (8, 17). Importantly, EndMT has been demonstrated to occur in both preclinical models of liver fibrosis (11, 18) and end-stage liver disease in patients (11, 12). The role of EndMT as a pathogenic driver, rather than a consequence of fibrogenesis, and its predictive value regarding disease progression remains ill-defined. We hypothesize that fibrosis-associated endothelial cells (FAEC) are EndMT-derived subpopulations that instigate both profibrotic and proinflammatory pathways, driving the early transition from tissue injury to progressive liver fibrosis.

To address our hypothesis, we developed a multi-omic approach integrating single-cell RNA-seq (scRNA-seq), immunofluorescence microscopy (IF) and full-spectrum flow cytometry (FSFC) to identify and phenotype EC subpopulations during different stages of carbon tetrachloride–induced (CCl_4_-induced) and Western diet– (WD-induced) mouse models of liver fibrosis. We characterize the induction of 2 distinct EndMT-derived FAEC populations in our preclinical studies and translate our findings to 3 cohorts of patients with MASLD. We show that TAGLN^+^ FAEC are a common immunomodulatory EC subpopulation that share transcriptional characteristics, arising in both mouse models and patient samples, that are significantly elevated at early-stage disease prior to clinical appearance of fibrosis. Furthermore, the elevated expression of *TAGLN* is a significant risk factor for liver decompensation events, even in the absence of fibrosis, and confers a significant additive effect for detrimental patient outcomes.

Taken together, we believe this study will pave the way for developing FAEC-specific diagnostic and therapeutic approaches to tackle progressive fibrotic liver disease.

## Results

### Mapping EC plasticity in healthy and fibrotic liver tissue.

We analyzed a publicly available dataset (GSE145086) enriched for hepatic immune cells, hepatic stellate cells (HSC) and EC in control C57BL/6 mice or following 2 and 4 weeks of CCl_4_-induced liver fibrosis (19) (Figure 1A). We identified 4 hepatic EC clusters that expressed combinations of genes known to be characteristic for each zone (4, 6): Zone 1 EC (Cluster 8), Zone 2 EC (Clusters 0 and 1), and Zone 3 EC (Cluster 6; Figure 1B). Each cluster also displayed distinct expression profiles for 5 EC-identity genes that are expressed as cell surface proteins: *Pecam1* (CD31), *Mcam* (MCAM), *Thbd* (TM), *Emcn* (EMCN), and *Lyve1* (LYVE1) (Figure 1, B and C). Immunofluorescence intensity profile for each of these markers, across the 200 μm distance between a portal and central vein, confirmed this zonal expression in healthy liver tissue (Figure 1, D and E).

After 4 weeks’ treatment with CCl_4_ to induce fibrosis, there was a shift in the abundance of midlobular EC populations during fibrogenesis (Figure 2, A and B), with pseudotime analysis showing reduction in the Zone 2 (Cluster 0) seen in health and expansion of what we have termed FAEC phenotype (Cluster 1) that transition toward Zone 1 and Zone 3 clusters (Figure 2C). These expanding FAEC display enriched expression of a panel of EndMT-associated, TGF-β–driven, genes after 4 weeks’ CCl_4_ treatment (Figure 2D). Notably, the expression of the EC identity genes also changes dynamically in response to CCl_4_ treatment (Figure 2D), highlighting the plasticity of hepatic EC during fibrogenesis.

To study these shifts in EC identity and phenotype, we treated male C57BL/6 mice with peanut oil vehicle alone (sham), CCl_4_ for 4 weeks (4w; early fibrosis) or 8w (established bridging fibrosis); alternatively, they were treated for an initial 4 weeks and then allowed to recover from tissue fibrosis for a further 4 weeks (Recovery Group; Figure 3, A and B).

Consistent with the transcriptional changes described above (Figure 2), quantification of widefield IF images revealed significant changes in vascular activation showing the increase in colocalization of vascular identity markers (CD31, TM, and EMCN; Figure 3C and Supplemental Figure 1, A–D; supplemental material available online with this article; https://doi.org/10.1172/jci.insight.207327DS1), together with TAGLN (Figure 3C and Supplemental Figure 1, A and E), resulting in a robust induction of TAGLN^+^ FAEC (Figure 3D), which were preferentially localized in Zone 3 pericentral regions (Figure 3E). To assess the influence of TGF-β isoforms and TGF-β receptor family members on the induction of TAGLN^+^ FAEC, we assessed the change in expression by qPCR, with the reduction in *Tgfbr2* at 8w CCl_4_ being the only significant difference at the transcriptomic level (Supplemental Figure 1F). In line with previous observations that *TAGLN* expression is preferentially induced by TGF-β2 (20–22), we show that TGF-β2 induced *TAGLN* expression with significantly greater efficacy compared with equivalent concentrations of TGF-β1 (Supplemental Figure 1G); in vivo, we assessed the proteomic expression of TGF-β2 in liver tissue (Supplemental Figure 1H). Profile plot analysis revealed a significant increase in TGF-β2, peaking in zone 3 (Supplemental Figure 1, I and J).

Taken together, we show that endothelial plasticity pathways are activated in distinct zones during liver fibrogenesis and tissue recovery, validating the suitability of this model to assess the role of EndMT in specific EC subpopulations leading to the derivation of FAEC phenotypes.

To observe phenotypic changes in multiple EC subpopulations simultaneously, a 14-marker panel was developed for FSFC (23), enabling us to profile the expression of 6 EC identity (CD31, MCAM, TM, EMCN, LYVE1, and PDPN), activation (CD34, ICAM1, and LY6A), and EndMT (TAGLN, THY1.2, and PDGFA) markers (Supplemental Figure 2A). All 4 treatment groups were concatenated, and CD31^+^CD45^–^ ECs (Supplemental Figure 2B) were clustered based on expression of the identity markers, producing 12 populations (Concatenated data; Figure 4A and split by treatment group Figure 4B) that were subcategorized each based on change in abundance: Reduced: (significantly reduced abundance following CCl_4_ treatment compared with Sham), Retained (no significant change following CCl_4_ treatment compared with Sham), and Expanded (significantly increased abundance compared with Sham), as quantified in Figure 4C. We show a striking alignment between the transcriptomic (Figure 2B) and proteomic (Figure 4C) plasticity data of hepatic EC but also highlight the considerable proteomic heterogeneity, which offers further insight into the phenotypic attributes of EC subpopulations during fibrogenesis and recovery.

### Phenotyping EC subpopulations during fibrogenesis and tissue recovery.

Reduced ECs were the predominant subcategory of EC in healthy liver, comprising 3 clusters (Clusters 1–3; Figure 5A), which become less abundant following 4w and/or 8w CCl_4_ and returned toward basal levels in the Recovery group (Figure 5, B–D). All 3 Reduced EC clusters are LYVE1^+^ICAM1^–^ and can be distinguished based on expression of TM and EMCN (Figure 5E), suggesting that the majority of these EC are situated in Zones 2 and 3 based on the expression profiles in Figure 1. These ECs had limited activation profiles, with LY6A induced in Cluster 2 only (Figure 5F) and no EndMT markers in response to CCl_4_ (Supplemental Figure 3, A–C). The key phenotypic characteristics of the clusters in this category are summarized in Figure 5G.

Five Retained clusters (Clusters 4–8; Figure 6A) did not change in abundance between healthy and CCl_4_-treated mice (Figure 6, B–F), although some clusters did express markers of activation and EndMT: MCAM^+^LY6A^+^TAGLN^+^ Cluster 4 and PDGFRα^+^ Cluster 6 (Supplemental Figure 3, D–H). Based on their expression profiles for Ly6A^+^ICAM1^–^ and differential expression of LYVE1 and EMCN (Figure 6, G–I), these EC do not appear to be associated to a specific zone.

Four Expanded EC clusters (Clusters 9–12; Figure 7A) significantly increased in abundance between healthy and CCl_4_-treated mice (Figure 7, B–E). We collectively define these clusters as FAEC, as they are instigated in response to tissue fibrosis while returning to basal levels in the Recovery group. All expanded clusters expressed elevated markers of activation and EndMT (Supplemental Figure 3, I–L). Notably, Clusters 9 and 10 expressed classical EndMT markers, downregulating the expression of the EC identity markers EMCN and TM, respectively, while inducing the expression of THY1.2/PDGFRα, with increased inflammatory activation via ICAM1 (Supplemental Figure 3, I and J). The most prevalent Expanded clusters, 11 and 12, displayed nonclassical EndMT phenotypes exhibiting both increased expression of EC identity markers, including CD31 and TM, together with enhanced TAGLN and LY6A expression (Supplemental Figure 3, K and L). The idiosyncrasies of these 4 clusters are further emphasized by the differential contour plots (Figure 7F) and histograms between the Sham and 8w CCl_4_ groups (Figure 7G), and they are summarized in schematics (Figure 7, H and I).

In 8w CCl_4_– and Sham-treated mouse liver sections, THY1.2^+^ and TAGLN^+^ FAEC are mutually exclusive cells within Zone 2 and Zone 3 regions of the liver, respectively (Figure 8, A and B). To determine whether TGF-β–induced EndMT can give rise to both FAEC populations, isolated healthy mouse liver EC stimulated in vitro with 10 ng/mL TGF-β2 for 48 hours also showed differential expression of THY1.2 and TAGLN (Figure 8, C and D).

Together, these data demonstrate EC heterogeneity and plasticity during fibrosis and tissue recovery and identify 2 distinct types of FAEC. While THY1.2^+^ and TAGLN^+^ FAEC may arise from a common TGF-β–driven EndMT pathway, they may also originate from separate healthy EC subpopulations. To test this assertion, we addressed the potential for Reduced clusters to transition toward Expanded FAEC clusters using Wishbone trajectory analysis.

### Derivation of FAEC using Wishbone trajectory mapping.

To determine whether EC in Reduced clusters transition toward Expanded FAEC, we first assessed their overlapping traits. Reduced clusters 1 and 3 and Expanded clusters 9 and 10 overlapped in expression of LYVE1 (Figure 9A) and LY6A (Figure 9B) in Sham animals and displayed marked plasticity following treatment. Notably, a subset of Cluster 10 became THY1.2^+^ (Figure 9B), while there was a complete transition for Cluster 9 toward a THY1.2^+^ phenotype in response to CCl_4_ treatment (Supplemental Figure 3, I and J). Wishbone trajectory analysis for the 4 clusters at all fibrosis stages, showed that LYVE1^hi^ICAM1^lo^THY1.2^lo^ EC converge at a branch point, which enables them to either shift toward a FAEC phenotype of clusters 9 and 10 in response to 8w CCl_4_ treatment or return to a healthy phenotype during recovery (Figure 9C). The inverse relationship of these markers is highlighted in the trajectory heatmaps showing that LYVE1 and THY1.2 appear to be mutually exclusive, while ICAM1 expression is enhanced at a later stage of transition to FAEC (Figure 9D). The putative transition pathway is summarized schematically (Figure 9E).

Similarly, Reduced Cluster 2 and the 2 TAGLN^+^ FAEC clusters 11 and 12 displayed overlapping expression of MCAM and LY6A (Figure 10A) and TAGLN (Figure 10B), which were all elevated following 4 and 8 weeks’ CCl_4_ treatment. Incremental acquisition of the MCAM expressing TAGLN^+^ FAEC phenotype during fibrosis is only partially restored after recovery, suggesting that these FAEC may persist in the tissue even after the insult is removed (Figure 10C). The trajectory heatmap highlights the persistence of TAGLN, LY6A, and MCAM in both the fibrogenic and recovery branches (Figure 10D), suggesting that these FAEC have decreased plasticity, impairing their capacity to recover a healthy phenotype (Figure 10E).

### Immunomodulatory function of FAEC.

To assess the influence of these 2 distinct FAEC subpopulations on the inflammatory response within the liver during fibrogenesis and recovery, we explored the transcriptomic signatures of EC enriched for either *Thy1* or *Tagln2* in the scRNA-seq dataset and compared them with the phenotypic characteristics in our FSFC data. Abundance of these cell types was similar: *Thy1*^+^ FAEC comprised ~1% of total EC by scRNA-seq and THY1.2^+^ FAEC ~5% of total EC by FSFC, while *Tagln^+^* (scRNA-seq) and TAGLN^+^ (FSFC) FAEC each comprised ~20% of total EC. Gene expression in *Thy1^+^* FAEC was indicative of leukocyte interaction and activation, (Figure 11A) and THY1.2^+^ FAEC expressed increased levels of ICAM1 following CCl_4_ treatment in both Cluster 9 and 10, along with reduced levels of the antiinflammatory glycoprotein EMCN in Cluster 9 only (Figure 11B). *Tagln^+^* FAEC displayed more specific enrichment for metabolic and inflammatory genes (Figure 12A) and TAGLN^+^ FAEC were characterized by upregulation of MCAM and LY6A (Figure 12B). In line with the localization and phenotype of FAEC, CD45^+^ leukocytes (Figure 12C) are preferentially recruited to pericentral regions following 4 and 8 weeks’ CCl_4_ treatment (Figure 12D).

To assess the inflammatory microenvironment induced by the different treatment groups, we profiled the abundance of CD45^+^CD31^–^ leukocyte subtypes using a 20-marker immune cell FSFC panel performed in parallel to the EC panel (Supplemental Figure 2A). These data describe a temporal-specific leukocyte profile initiated by CCl_4_, which peaks in the 4w CCl_4_ group (Supplemental Figure 4A) with significant elevation of dendritic cells, B cells, NK cells, and F4/80^+^ macrophages (Supplemental Figure 4, B–E), with no significant changes noted for recruited monocytes, mature macrophages, neutrophils, or T cell subtypes (Supplemental Figure 4, F–J).

CCl_4_ is a well-established hepatoxic substance, inducing apoptosis and necrosis in Zone 3 hepatocytes (24, 25), which instigate immune cell recruitment. To determine the contribution of TAGLN^+^ FAEC to the infiltration of CD45^+^ leukocytes, we compared the colocalization of CD45^+^ leukocytes with TAGLN^+^ FAEC and markers of apoptosis (cleaved caspase-3) and necrosis (RIPK3). By imaging sequential sections, we show that, while all 3 cell types are significantly induced by CCl_4_ treatment, they are distinct from each other (Supplemental Figure 5A). They show different profiles with TAGLN^+^ FAEC peaking in the 4w CCl_4_ treatment group, while apoptosis and necrosis peaked in the 8w CCl_4_ group (Supplemental Figure 5B). Notably, by imaging CD45^+^ leukocytes with each cell type (Supplemental Figure 5C), we could quantify the percentage of leukocytes associated with TAGLN^+^ FAEC, cleaved caspase-3^+^ apoptotic cells, and RIPK3 necrotic cells revealing that the most significant interactions occur with TAGLN^+^ FAEC in the 4w CCl_4_ (Supplemental Figure 5, D–F).

### TAGLN^+^ FAEC are conserved in WD-fed mice and patients with MASLD.

Having characterized the induction of FAEC subpopulations in a murine CCl_4_-induced liver fibrosis, we sought to determine if FAEC were also an early characteristic of a murine dietary model of MASLD induced by feeding animals a WD. There was significant uptake of lipid within the liver (Figure 13, A and B) and moderate hepatic fibrosis (Figure 13, C and D). In keeping with the instigation of mild tissue fibrosis, compared with the CCl_4_ model, the effect on vascular architecture (Supplemental Figure 6, A and B) and EC identity markers was less pronounced but still resulted in significantly increased EMCN, TM, and TAGLN following 12w of WD (Supplemental Figure 6C).

Assessment of EC subpopulations by FSFC distinguished 12 hepatic EC clusters (Figure 13E). Consistent with our earlier findings (Figure 4A), we identified phenotypically distinct Reduced, Retained, and Expanded subpopulations (Supplemental Figure 7, A–L), though the differences in proportion were more subtle than CCl_4_ (Figure 13F). Reduced clusters 1 and 2 (Figure 13G) and Expanded clusters 11 and 12 (Figure 13H) shared overlapping characteristics, such as ICAM1 and TAGLN expression, when compared on the UMAP (Figure 14, A and B). Wishbone analysis revealed a shared trajectory toward a TAGLN^+^ FAEC phenotype (Figure 14, C and D), which occurred more gradually than in response to CCl_4_ (Figure 14E).

The immune profile of the 12w WD showed no significant difference in total CD45^+^ numbers (Supplemental Figure 8A) but a dynamic shift in specific subtypes with a significant increase in dendritic cells, total macrophages, recruited monocytes, and mature macrophages (Supplemental Figure 8, B–E) with no observable changes in the other leukocyte populations assessed (Supplemental Figure 8, F–J). To assess the ability of TAGLN^+^ FAEC to interact with leukocytes in this model, we imaged CD45 in combination with TAGLN^+^ FAEC, apoptosis, and necrosis (Supplemental Figure 8M). Unlike the CCl_4_ treatment group (Supplemental Figure 5), 12w WD induced a significant increase in TAGLN^+^ FAEC without observable increase in CD45^+^ leukocytes or expression of cleaved caspase 3^+^ apoptotic or RIPK3^+^ necrotic cells (Supplemental Figure 8, N–P).

Finally, we examined whether the induction of TAGLN expression was associated with disease severity and patient outcomes in human MASLD. In liver biopsy samples from patients who underwent bariatric surgery, there was a significant expansion of both the number of *TAGLN*^+^ FAEC and expression of *TAGLN*^+^ in patients with fibrosis compared with those without fibrosis (Supplemental Figure 9A). IF staining for TAGLN^+^CD31^+^ EndMT cells (Supplemental Figure 9B) showed a significant increase in patients with early stages (F0/1) of disease (Supplemental Figure 9C) compared with healthy control. In keeping with the phenotype of TAGLN^+^ FAEC in murine models, we found enrichment of *MCAM*, *TAGLN*, and *THY1* (Supplemental Figure 9D) with GO analysis showing similar enrichment to the mouse data in vascular remodeling, profibrotic, and proinflammatory pathways (Supplemental Figure 9E). We show that MCAM^+^ expressing TAGLN^+^ FAEC are the most prominent phenotype in the 2 mechanistically distinct models of murine liver fibrosis and are expanded in patients who have undergone bariatric surgery (Supplemental Figure 9F).

In spatial transcriptomic analysis of 4 patient biopsies (Supplemental Table 1), we show that TAGLN^+^ FAEC colocalize within ECM and inflammatory macrophages with regions of fibrosis (Figure 15A). In line with patient scRNA-seq data, the appearance of TAGLN^+^ FAEC was apparent at early-stage disease before observable fibrosis and persisted in patients with cirrhosis (Supplemental Figure 10A). Notably, localization of TAGLN^+^ FAEC was closely associated with the expression of TGF-β isoforms and TGF-β receptor (TGF-βR) family members (Figure 15, B and C). Comparison of TAGLN^+^ FAEC versus TAGLN^–^ EC transcriptomic signatures revealed that profibrotic (Collagen) and proinflammatory (ICAM) pathways were preferentially enriched in TAGLN^+^ FAEC (Figure 15D and Supplemental Figure 10B). Detailed CellChat analysis identified the expression of *THBS*, *LAMA*, and *COL* as potentially unique sender/receiver partners (Figure 15E and Supplemental Figure 11, A and B).

To evaluate the potential clinical effect of elevated TAGLN expression in patients with MASLD, we utilized the SteatoSITE (26) database to assess patient outcomes. High *TAGLN* expression (above 26.76 cpm) in liver biopsies revealed patients at a significantly elevated risk of all-cause mortality compared with those with lower expression levels (Figure 16A). Furthermore, patients with high levels of *TAGLN* but no fibrosis (Figure 16B, F0; green line) had a significantly higher risk of hepatic decompensation than patients with cirrhosis but low *TAGLN* expression (Figure 16B, F4; blue line), suggesting that *TAGLN* expression has the potential to be an early and fibrosis-independent risk factor of detrimental patient outcomes.

Overall, our data elucidate the derivation of 2 distinct FAEC subpopulations by integrating scRNA-seq and IF with a novel FSFC analysis of EC plasticity. Notably, these FAEC share common profibrotic and proinflammatory phenotypes, which have potential immunomodulatory functions by colocalizing with leukocytes (Supplemental Figure 11C). Finally, we reveal that the induction of *TAGLN* expression is significantly associated with detrimental clinical outcomes in patients with liver disease, independent of fibrosis severity, supporting the hypothesis that TAGLN^+^ FAEC play an underappreciated role in disease pathogenesis.

## Discussion

The role of vascular heterogeneity in health and disease has come into sharp focus in recent years, with considerable advances in single-cell transcriptomic (7, 27) and proteomic (6) profiling revealing remarkable insight into organotypic and pathological endothelial heterogeneity. In this study, 3 broad categories of liver EC in liver fibrosis: Reduced, Retained, and Expanded have been defined and phenotyped. We find that the Reduced clusters that express CD31^+^LYVE1^+^MCAM^–^ (some also expressed EMCN) localize to midlobular (Zone 2) and pericentral (Zone 3) regions, the same regions where the Expanded cells arise from, in keeping with known CCl_4_ damage pattern (28, 29). Using Wishbone trajectory analysis, we were able to show that they can change their phenotype and contribute to the development of Expanded populations. All Expanded clusters expressed EndMT traits as well as enhanced inflammatory profiles in response to CCl_4_-induced liver fibrosis. These EC clusters were the most highly plastic of all the EC subpopulations assessed, dramatically changing their abundance and phenotype in response to both CCl_4_ time points and following tissue recovery; thus, we designated them as FAEC. Finally, the association of these markers with diseases severity and outcome in human disease strongly suggest translational potential of these findings (30).

EC are highly plastic (31) and their regulation of reparative or pathogenic pathways (32, 33) have diagnostic and therapeutic potential in human disease. We and others have determined that TGF-β–induced EndMT cells are an important component of the fibrogenic hepatic vascular niche (11, 12, 18, 34); however, the functional consequences of these phenotypic changes remain elusive. There is currently no consensus on how to define EC heterogeneity in the liver, and the application of multiple techniques has led to a variety of distinct subpopulations, ranging from IHC studies (35) detailing type 1 and type 2 LSEC to scRNA-seq identifying 5 to 7 endothelial populations in mice (36) and humans (10). A key advancement of our study is the complementary use of available scRNA-seq data with multiple identity marker and high-throughput proteomic phenotyping through FSFC. While previous studies applied lineage tracing approaches to validate the occurrence of EndMT (7, 12, 17, 37) in several tissues and disease states, they are restricted to an individual identity marker. In contrast, our approach provides a more comprehensive analysis of EC plasticity, giving large-volume, single-cell data with multiplexing capabilities, high-dimensional reduction analysis, and predictive algorithms, such as Wishbone trajectory (23, 38), to trace the derivation of FAEC populations.

The most prominent distinguishing feature of these FAEC clusters was the expression of either THY1.2^+^ or TAGLN^+^. THY1.2^+^ FAEC express higher levels of ICAM1 and do not express EMCN and LYVE1. THY1.2 is a member of the immunoglobulin supergene family and is commonly associated with smooth muscle cells. EC expressing *Thy1* are associated with inflammation-induced angiogenesis (39) and transendothelial leukocyte migration (40). ICAM1 is a key regulator of leukocyte rolling and adhesion to LSEC (41). Conversely, loss of EMCN and LYVE1 can also influence immune cell interactions, as both are documented to play protective roles in preventing aberrant leukocyte recruitment, respectively (42, 43).

TAGLN^+^ FAEC were not only the most abundant but were also conserved across species and displayed reduced plasticity, which may account for their persistence across all stages of liver disease assessed. TAGLN, a member of the calponin superfamily, is an actin-binding protein that can regulate the cytoskeleton (44), and ectopic TAGLN expression is hallmark of EndMT (31, 45). The upregulation of MCAM may point toward its role as an adhesion molecule that promotes cell-ECM and cell-cell interactions (46). MCAM is a well-characterized biomarker for advanced liver disease (47), and it is known to induce procoagulant responses in metastatic tumors (48). Upregulation of Ly6A is consistent with previous reports detailing the expansion of Ly6A^+^ LSEC during liver injury (49, 50). Notably, high expression of MCAM in TAGLN^+^ FAEC is a conserved phenotype that is induced in early stages of liver injury in both CCl_4_- and 12-week WD–induced mouse models and in patients without observable fibrosis, suggesting that the appearance of TAGLN^+^ FAEC could be a novel, sensitive marker of disease progression.

TAGLN^+^ FAEC colocalize with regions enriched for ECM and infiltrating leukocytes in both mouse and human fibrosis. Within our preclinical models of liver fibrosis, we show that TAGLN^+^ FAEC are distinct from apoptotic and necrotic hepatocytes and are the predominant interacting cell type with CD45^+^ leukocytes at early stages of tissue injury. The spatial transcriptomics CellChat DB analysis revealed a notable enrichment of CD44 ligands on TAGLN^+^ FAEC, which provides insight to assess whether TAGLN^+^ FAEC preferentially interact with specific immune subtypes in future studies. Importantly, CD44-deficient mice are protected in mouse models of MASH (51) while upregulation of CD44 expression is linked to both disease severity (51) and the transition to hepatocellular carcinoma (52), which further implicates the detrimental role of TAGLN^+^ FAEC in the progressive disease.

To investigate the clinical implications of aberrant *TAGLN* expression, we interrogated the large SteatoSite cohort of histologically confirmed MASLD (26). We observed that high hepatic *TAGLN* expression is significantly associated with adverse clinical outcomes, which is in line with studies in other pathologies that have linked *TAGLN* expression with poor prognosis in pancreatic (53) and colorectal (54) cancers. By separating the patient data by fibrosis stage, we show that elevated TAGLN expression is a sensitive biomarker of liver decompensation events, independent of fibrosis, which could offer prognostic value for identifying patients at risk of progressive liver disease.

Taken together, we have identified distinct EndMT-derived FAEC with defined localization and characteristics that have the capacity to reshape the vascular and immune landscape of the liver. TAGLN^+^ FAEC represent an exciting opportunity to develop diagnostic and therapeutic approaches to tackle the growing clinical burden of chronic liver disease.

## Methods

### Sex as a biological variable.

Sex was not considered as a biological variable in this study. All animal experiments were performed using male C57BL/6 mice. The decision to use male animals was based on the established use of this sex in the CCl_4_ and WD models of liver fibrosis, where hepatic injury and fibrogenic responses are well characterized in males and provide a consistent and reproducible experimental framework. Whether the FAEC populations and EndMT responses identified here are equivalent in female mice remains to be determined in future studies. With respect to human data, the SteatoSITE cohort and bariatric surgery patient cohorts included both male and female participants, and sex was included as a covariate in the SteatoSITE survival and time-to-event analyses. The spatial transcriptomic patient cohort included both male and female individuals (Supplemental Table 1), though the small sample size precluded sex-stratified analysis.

### Animals.

Eight- to 10-week-old WT C57BL/6 male mice were purchased from Charles River Laboratories UK Ltd. (Kent, UK). For all in vivo experiments, power calculations were performed using preliminary data to calculate the appropriate number of animals to be included in the study.

### Murine models of liver injury.

CCl_4_ (289116-100ML, Sigma) was diluted 1 in 5 in peanut oil (P2144-250ML, Sigma) and was administered with an i.p. injection at a dose of 0.8 mg/kg in 100 μL. Injections were administered twice a week for 2, 4, or 8 weeks. For the recovery experiments, mice were injected with CCl_4_ for 4 weeks and were afterward left to recover for an additional 4 weeks. As a control, no treatment or Sham injections of 100 μL peanut oil were used.

To model a WD and induce hepatic steatosis and fibrosis, mice were fed the modified Clinton-Cybulsky cholesterol diet (58R6, IPS Product Supplies) with the addition of carbohydrates (42 g/L) consisting of 55% fructose (F0127-1KG, Sigma-Aldrich) and 45% sucrose (S9378-1KG, Sigma-Aldrich) in the drinking water for 12 weeks. At each stated time point, animals were sacrificed, followed by cardiac perfusion with 3 mL sterile deionized phosphate-buffered saline (PBS) (BR0014G, Thermo Fisher Scientific) with 0.5% bovine serum albumin (BSA) (BP9702-100, Thermo Fisher Scientific) and 2 mM EDTA (E7889-100ML, Sigma) through the LV. Individual liver lobes were removed for IHC, tissue digestion for tissue culture or flow cytometry, and RNA extraction.

### IHC.

Tissues were fixed for 2 hours in 4% paraformaldehyde before being prepared for either OCT or paraffin embedding. Tissue sections were cut at 10 μm or 5 μm, respectively. Samples were initially blocked in 3% BSA diluted in PBS + 0.1% Tween 20 (P1379-100ML, Sigma) (PBST) for 1 hour at room temperature (RT). The samples were incubated with primary antibodies (Supplemental Table 2) overnight (diluted in 1% BSA). They were then washed 3 times with PBST and incubated with appropriate secondary antibodies (Supplemental Table 2) for 1 hour at RT. The slides were washed and mounted with FluoroMount G (00-4958-02, Invitrogen). Images were acquired using an inverted confocal laser scanning microscope at a 10× magnification.

### Picrosirius red staining.

Paraffin-embedded sections were deparaffinized with Histoclear solution (NAT1330, Scientific Laboratory Supplies) and rehydrated. For staining, the Abcam Picrosirius red stain kit was used (ab245887, Abcam). The slides were then incubated in phosphomolybdic acid for 8 minutes and were transferred to picrosirius red stain for 60 minutes. They were then washed 3 times with acetic acid and were mounted using the Entellan (107960, Merck) mounting medium. The samples were visualized using the Olympus CKX53 light microscope (Olympus, Japan), and images were acquired at 4× magnification.

To analyze the data, the whole region from 2 sections per sample was quantified. The percentage (%) of picrosirius red^+^ area was calculated using ImageJ (NIH) and the color deconvolution macro (55).

### Cell culture of human umbilical vein endothelial cells.

Commercially available HUVEC were obtained from PromoCell (C-12203). Cells were seeded in wells of 6- or 12-well plates at a density of 200,000 or 80,000, respectively. The culture media used was the Lonza EBM2 Basal Medium (CC-3156, Lonza) supplemented with the EGM2 SingleQuots supplements (CC-4176, Lonza).

### Tissue digestion.

Liver samples were mechanically disrupted until homogenized using scissors. The homogenates were then further digested enzymatically in 3 mL digestion buffer containing Hank’s Balanced Salt Solution (HBSS) with CaCl_2_ and MgCl_2_ (14025092, Gibco) with 20 mM N-2-hydroxyethylpiperazine-N’-2-ethanesulfonic acid (HEPES) (15630-080, Gibco), 2 mg/mL collagenase (17018-029, Gibco), 8 U/mL dispase II (04942078001, Roche), and 50 U/mL DNase (18047-019, Invitrogen). For digestion, the homogenates were incubated at 37°C for 30 minutes on a shaker. The samples were then filtered twice through a 70 μm cell sieve (431751, Scientific Laboratory Supplies). The cell pellets were then resuspended for tissue culture or FSFC.

### Staining for FSFC.

Single-cell suspensions were transferred to a V-shaped 96-well plate (50 μL/well). The cells were washed with 200 μL PBS and were resuspended in 50 μL Fc blocking solution (130-092-575, Miltenyi Biotec) containing the Live/Dead Fixable Blue stain (L23105, Invitrogen) at a dilution of 1 in 5,000 for 30 min at 4°C. The cells were washed twice with 200 μL PBS and were then incubated with cell surface binding antibodies for 30 min at 4°C. FSFC primary antibody details are listed for the endothelial cell panel (Supplemental Table 3) and immune cell panel (Supplemental Table 4). The samples were then washed with PBS and centrifuged at 400*g* for 2 minutes at 4°C. For fixation and permeabilization, the cell pellets were resuspended in the intracellular fixation and permeabilization buffer (00-5521-00, Invitrogen), and the plate was incubated for 20 min at RT. The samples were then washed with 200 μL permeabilization wash buffer (00-8333-56, Invitrogen) and centrifuged at 400*g* for 2 minutes. The cell pellets were resuspended in the intracellular binding antibody solution and incubated for 30 minutes at RT. The samples were washed with permeabilization wash buffer, followed by centrifugation at 400*g* for 2 minutes. The cell pellets were resuspended in 200 μL PBS with 2% FBS (A4766801, Gibco) and 2 mM EDTA and were stored at 4°C for acquisition. Appropriate fluorescence minus one (FMO) controls were also prepared in parallel. For comprehensive details regarding this protocol refer to Gkantsinikoudi et al. (23).

### FSFC data acquisition and analysis.

FSFC data acquisition was performed using the Cytek 4 laser Aurora (UV, V, B, R) spectral flow cytometer and the SpectroFlow 3.3.0 software (Cytek Biosciences, US). For spectral unmixing, single-stain reference control beads were used for all fluorescent antibodies. Heat-killed cells (90°C) were used to prepare the reference control for the Live/Dead Fixable Blue stain. Multiple autofluorescence extraction was used to account for the autofluorescence signatures of different cell populations. For this, a tissue- and treatment-specific unstained sample was used.

All data were analyzed using the OMIQ Software (Dotmatics, US). Cellular debris was excluded from the analysis by appropriate gating using the FSC-A versus SSC-A plot, followed by exclusion of duplets using the FSC-A versus FSC-H plot. Live cells were selected based on the Live/Dead Fixable Blue stain. Then, in the CD45 versus CD31 plot, the CD45^+^ and CD45^–^ populations were analyzed separately. In the CD45^–^ gate, EC populations were analyzed, while in the CD45^+^ gate, analysis of immune cell populations was performed. Both classical gating analysis and dimensionality reduction analysis was used.

For dimensionality reduction, the first step of the analysis pipeline involved data quality control, which was performed using the FlowAI algorithm (56). Then, manual compensation was performed where necessary. For unsupervised clustering, the FlowSOM algorithm (57) and the cells were clustered based on the endothelial markers CD31, TM, EMCN, LYVE1, MCAM, and PDPN to generate endothelial clusters with different identities. For cluster visualization, uniform manifold approximation and projection for dimension reduction (UMAP) plots (58) were used. UMAP were generated using the Euclidean metric composed of the same 6 EC markers with a minimum distance of 0.1, nearest neighbors of 15. Contour plots, histograms, and Wishbone trajectory analysis were generated using OMIQ software as detailed (23).

### Image analysis and profile plot generation.

For image analysis, the Volocity 3D Image analysis software (PerkinElmer, US) was used. The total area for different markers was calculated and normalized using the total tissue area or total number of nuclei. For EndMT quantification, the area of colocalization of an endothelial and a mesenchymal marker was calculated.

To visualize and quantify the distribution of different markers across the 3 zones of the liver tissue, profile plots were generated using ImageJ and the Macro plot line profile multicolor macro written by Kees Straatman, University of Leicester (59), which was modified by Paul Imbert, CMR Advanced Bioimaging Facility, William Harvey Research Institute, Queen Mary University of London, London, United Kingdom. Regions of interest were selected that spanned from a portal vein (zone 1) led to a central vein (zone 3; Supplemental Figure 1A). A 200 μm line was used to measure the fluorescence intensity between these 2 structures. The distance between the portal and central veins was expressed as a percentage and segmented into zone 1 (0%–33% of line), zone 2 (34%–66% of line), and zone 3 (67%–100% of line) for normalization across all samples. Area under the curve was used to calculate the fluorescence signal under the curve within each zone.

### Single-cell RNA-seq data acquisition (mouse CCl_4_ study).

Raw 10x Genomics single-cell RNA-seq data from the GSE145086 dataset were obtained from the Gene Expression Omnibus and processed in R. For each sample, the standard 10x output files (gene-cell count matrix, barcode file, and gene annotation file) were imported, and sparse matrices were constructed alongside their corresponding barcodes and gene identifiers. Gene symbols were inspected for redundancy and made unique where necessary, and each cell was assigned a unique sample specific identifier to enable accurate provenance tracking. Per cell metadata were generated for every sample, and individual datasets were converted into Seurat objects. Following quality checks to ensure alignment of barcodes, metadata, and gene features, all samples were merged into a single unified Seurat object.

For trajectory reconstruction, Seurat objects were converted into Monocle3 cell data sets using the as.cell_data_set function from SeuratWrappers. Cluster and partition information from Seurat were retained. The principal graph was learned using learn_graph, and pseudotime was estimated using order_cells, setting the endothelial cell cluster representing the quiescent state as the root.

Pseudotime values were extracted and used to infer lineage progression among endothelial subpopulations. Only Monocle3’s internal pseudotime calculations were used for subsequent analysis; graphical visualization was performed in R but excluded from final interpretation.

Genes significantly upregulated in selected comparisons were converted to Entrez IDs using the bitr function in the clusterProfiler (v4.10.0) package (OrgDb = org.Mm.eg.db). Gene ontology (GO) enrichment analysis for Biological Processes (BP) was performed using enrichGO (with the Benjamini-Hochberg adjusted *P* value threshold of 0.05). The most significantly enriched BP were used for interpretation of functional differences among endothelial subpopulations.

All analyses were conducted in R (v4.3.3) on Apocrita HPC system (https://zenodo.org/records/438045). The following packages were used: Seurat (v5.0.3), Monocle3 (v1.3.7), SeuratWrappers (v0.3.1), clusterProfiler (v4.10.0), org.Mm.eg.db (v3.17.0), ggplot2 (v3.5.1), and dplyr (v1.1.4).

### Marker gene identification and differential expression.

Cluster-specific marker genes were identified using Seurat’s FindAllMarkers function, retaining genes expressed in at least 25% of cells within a cluster and exhibiting a log fold change greater than 0.25. Differential expression analyses between experimental conditions were performed using FindMarkers with test.use = “MAST”, and significant genes were defined by adjusted *P* < 0.05 and log_2_FC > 0.25.

All results were exported as CSV files for reproducibility and integration with pathway enrichment analysis.

### CiteSeq sequencing patients who have undergone bariatric surgery.

We performed antibody-augmented single-cell RNA-seq for cell populations from liver tissue obtained during bariatric surgery (liver disease) or gallbladder surgery (healthy). Histological assessment of MASLD and fibrosis was conducted by a specialist pathologist according to the NASH CRN. Log-normalized TAGLN expression values were exported from the Seurat object and plotted in GraphPad Prism to compare expression between healthy and fibrotic liver samples (60).

### High-definition spatial transcriptomics data generation and analysis.

Patient biopsy FFPE sections (5 μm) were prepared and stained according to the Visium HD FFPE Tissue Preparation protocols (GC000684, Revision D) and processed following the standard Visium HD Spatial Gene Expression Reagents Kits User Guide (GC000685, Revision D) as outlined by 10x Genomics. Prepared libraries were sequenced on the Illumina NovaSeq 6000 SP lane with 100 cycles. The sequencing data were preprocessed using Space Ranger (v3.1.3, 10x Genomics), including the generation of the CLOUPE output at 8 × 8 μm binning resolution. On a per-biopsy basis, endothelial bins were identified within Loupe Browser (v.8.0.0, 10x Genomics) by a Log2 Feature Sum of endothelial marker genes (PECAM1, THBD, EMCN, MCAM, LYVE1) and split into TAGLN^+^ FAEC and TAGLN^–^ Liver ECs. Nonendothelial bins were defined as “rest_of_tissue”. Differential gene expression between TAGLN^+^ FAEC and Liver EC-associated bins was identified within Loupe Browser using the “Run Differential Expression” tool, with significance adjusted for multiple comparisons using Benjamini-Hochberg correction. To define potential receptor-ligand analyses, barcode annotations were exported from Loupe Browser. The Visium HD raw counts data matrix was loaded into a SpatialData object, subset to the relevant barcodes prior to annotation transfer, normalization, and log transformation of the expression data and h5ad conversion. CellChat (v2.2.0) was used to generate a spatial CellChat object for each biopsy using the full human interaction database (CellChatDB.human) and to generate visualizations. Compute CommunProb was performed using parameters “type = truncated Mean” and “trim=0.01”.

### SteatoSITE analysis.

SteatoSITE v2 with follow up to end December 2023 was used. Only biopsy samples with RNA-seq were used for analysis using R4.4.1. For overall survival analysis (*n* = 505), surv_cutpoint() from the “survminer” (0.5.1) with a minimum proportion 0.25 was used to define the optimal cutpoint of TAGLN expression (normalized cpm) using the maximally selected rank statistics from the maxstat package (v. 0.7-26). For time-to-liver related event analysis, samples with no liver event coding prior to the biopsy date (*n* = 435) were fit with a time-to-event proportional hazards regression model using NASH CRN fibrosis stage, age, sex, and TAGLN expression as predictive variables using coxph from “survival” (3.6-4), and the cumulative incidence function of the model at t = 4,000 days stratified for NASH CRN fibrosis stage plotted as a function of TAGLN expression using a plot_surv_at_t from “contsurvplot” (0.2.2).

### Statistics.

All statistical analysis was completed using the GraphPad Prism 10.0.0 software (GraphPad, US). Statistical tests were performed to identify statistically significant results. The Shapiro-Wilk normality test was first used to identify whether the data are distributed normally. After that, a 2-tailed *t* test or a 1-way ANOVA was used for normally distributed data, with the Mann-Whitney *U* test or Kruskal-Wallis test used as the nonparametric equivalent where normality was not met. Appropriate multiple-comparison tests were also applied to all datasets. The exact test used for each dataset is detailed in the corresponding figure legend of each Figure. Statistical significance was defined as *P* ≤ 0.05. All data are presented as mean ± SEM.

### Study approval.

All procedures used in this study were approved by the Animal Use and Care Committee of Queen Mary University of London and were in accordance with national and international regulations (Project license: PP0505769).

Participants provided written informed consent at enrollment. The studies were approved by local ethics Committee (reference nos. 18/LO/1759, 14/WA/1142) and performed in compliance with the Declaration of Helsinki.

Anonymized tissue was supplied with approval from the National Health Service Research Scotland (NRS) Biorepository Network (Ref: SR1032; 2 Aug 2018). Unified transparent approval for unconsented data inclusion was granted by the West of Scotland Research Ethics Committee 4 (Ref: 25/WS/0004; 16 Jan 2025), the Public Benefit and Privacy Panel for Health and Social Care (Ref: 2425-0221; 20 May 2025), institutional R&D departments, and Caldicott Guardians.

### Data availability.

Murine scRNA-seq data were analyzed from 10x Genomics single-cell transcriptomic data of isolated nonparenchymal liver cells from healthy and diseased (2w and 4w CCl_4_ treated) mice deposited on the GEO database (GSE145086).

For access to the full SteatoSITE resource, including rich clinical outcome annotation, visit https://regeneration-repair.ed.ac.uk/research/research-resources/steatosite Raw RNA-seq data (FASTQ files) and minimal metadata (fibrosis stage) are available in the European Nucleotide Archive (https://www.ebi.ac.uk/ena; study accession no. PRJEB58625). Accession number for the Citeseq (Supplemental Figure 6) is available on the GEO databases GSE344087. Visium HD Spatial Transcriptomics analyses (Figure 8 and Supplemental Figure 7) is available on the GEO databases GSE334308. All data used to produce graphical panels in the manuscript are available in the Supporting Data Values file.

## Author contributions

Conceptualization was contributed by CG, JPD, WA, and NPD. Methodology was contributed by CG, RK, EJ, SL, TJK, and NPD. Investigation was contributed by CG, JPD, RK, EJ, MR, TJK, WL, MS, and NPD. Visualization was contributed by CG, RK, EJ, MR, TJK, and NPD. Funding acquisition was contributed by KPH, AR, WA, and NPD. Project administration was contributed by AR, JAF, SL, VSA, KPH, WA, and NPD. Supervision was contributed by AR, JAF, KPH, WA, and NPD. Writing of the original draft was contributed by CG, JPD, and NPD. Review and editing were contributed by CG, JPD, RK, EJ, TJK, WL, MS, AR, JAF, KPH, WA, and NPD.

## Conflict of interest

JAF would like to disclose that he serves as a consultant and/or advisory board member for Medixci, Resolution Therapeutics, Kynos Therapeutics, Gyre Therapeutics, Ipsen, River 2 Renal Corp., Stimuliver, Guidepoint, and ICON plc; has received speakers’ fees from HistoIndex, Resolution Therapeutics, and Société internationale de développement professionnel continu Cléo; and has received research grant funding from GlaxoSmithKline and Genentech.

## Funding support

- Medical Research Council project grant MR/Y013751/1 (ND).
- Bart’s Charity Seed Grant MGU0514 (ND).
- British Heart Foundation PhD studentship FS/4yPhD/F/21/34161 (CG).
- Medical Research Council project grant MR/T031883/1 (WA).
- Innovate UK project grant TS/U00457X/1 (KPH).
- Innovate UK project grant TS/R017581/1 (JAF/TJK).
- Guts UK DGO2019_16 (JAF/TJK).

## Supplementary Material

Supplemental data

Supporting data values

## Figures and Tables

**Figure 1:**
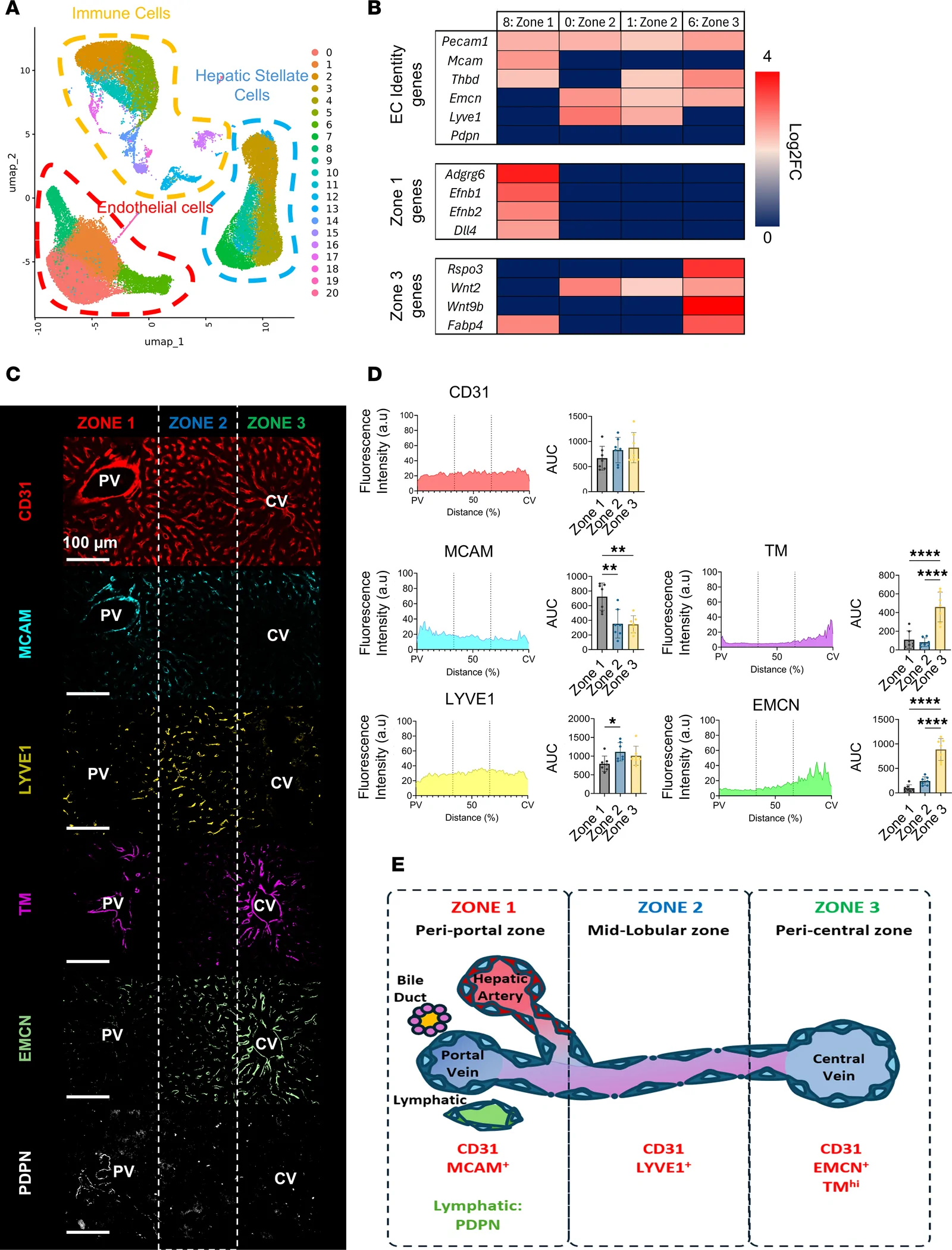
Characterization of EC identity and zonation in healthy tissue. (**A**) UMAP of nonparenchymal cell scRNA-seq data (GSE145086) for control (untreated), 2-, and 4-week CCl_4_-treated male C57BL/6 mice (19). (**B**) Heatmap of gene expression for the 4 EC clusters (clusters 0, 1, 6, and 8) under baseline conditions identifying the zonation of EC identity markers (top panel) based on expression of established markers for periportal /Zone 1 (mid-panel) and pericentral/Zone 3 (bottom panel) EC. (**C**) Representative confocal images of healthy liver tissue stained for the EC identity markers CD31, melanoma cell adhesion molecule (MCAM), LYVE1, thrombomodulin (TM), and endomucin (EMCN). Podoplanin (PDPN) included as lymphatic marker. (**D**) Immunofluorescence intensity distribution across the portal-central vein axis and bar graphs of signal quantification for each zone for CD31, MCAM, LYVE1, TM, and EMCN. Scale bars: 100 μm. (**E**) Schematic summarizing the zonal distribution of hepatic EC markers. *P < 0.05, **P < 0.01, ****P < 0.0001.

**Figure 2:**
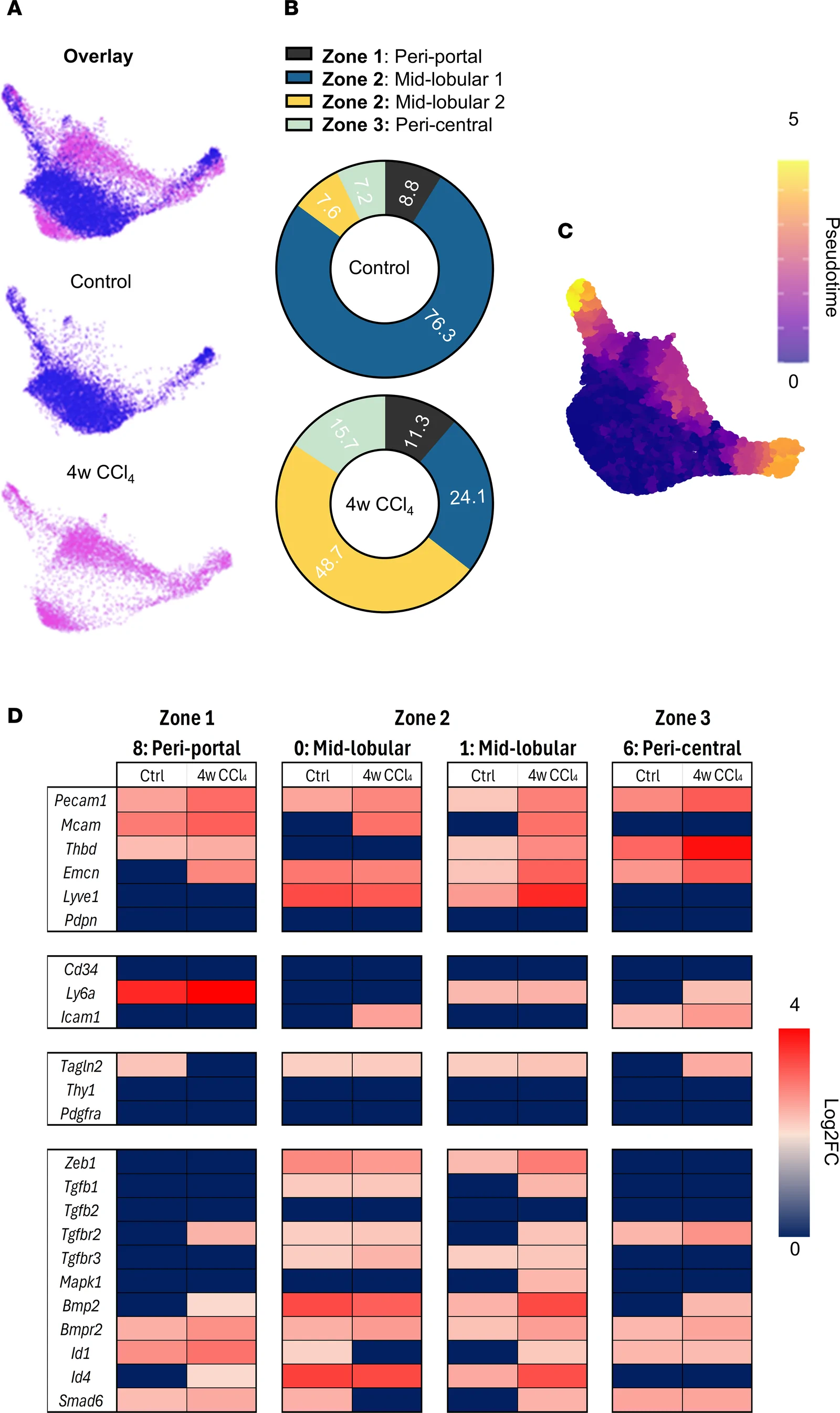
Characterization of EC identity and plasticity markers in fibrogenesis. (**A**) Visual overlay of EC clusters from scRNA-seq for control mice (blue) and 4w CCl_4_ treatment (magenta). (**B**) Quantification of the abundance of each EC cluster in control (top panel) and 4w CCl_4_ treated mice (bottom panel). (**C**) Pseudotime analysis of progressive fibrogenesis from healthy, 2w, and 4w CCl_4_–treated samples showing the transition away from cluster 0 mid-lobular EC toward clusters 1, 6, and 8. (**D**) Heatmap of gene expression for EC markers (top panels) and TGF-β–activated genes (bottom panels) for the 4 EC clusters comparing control and 4w CCl_4_ treatment groups.

**Figure 3:**
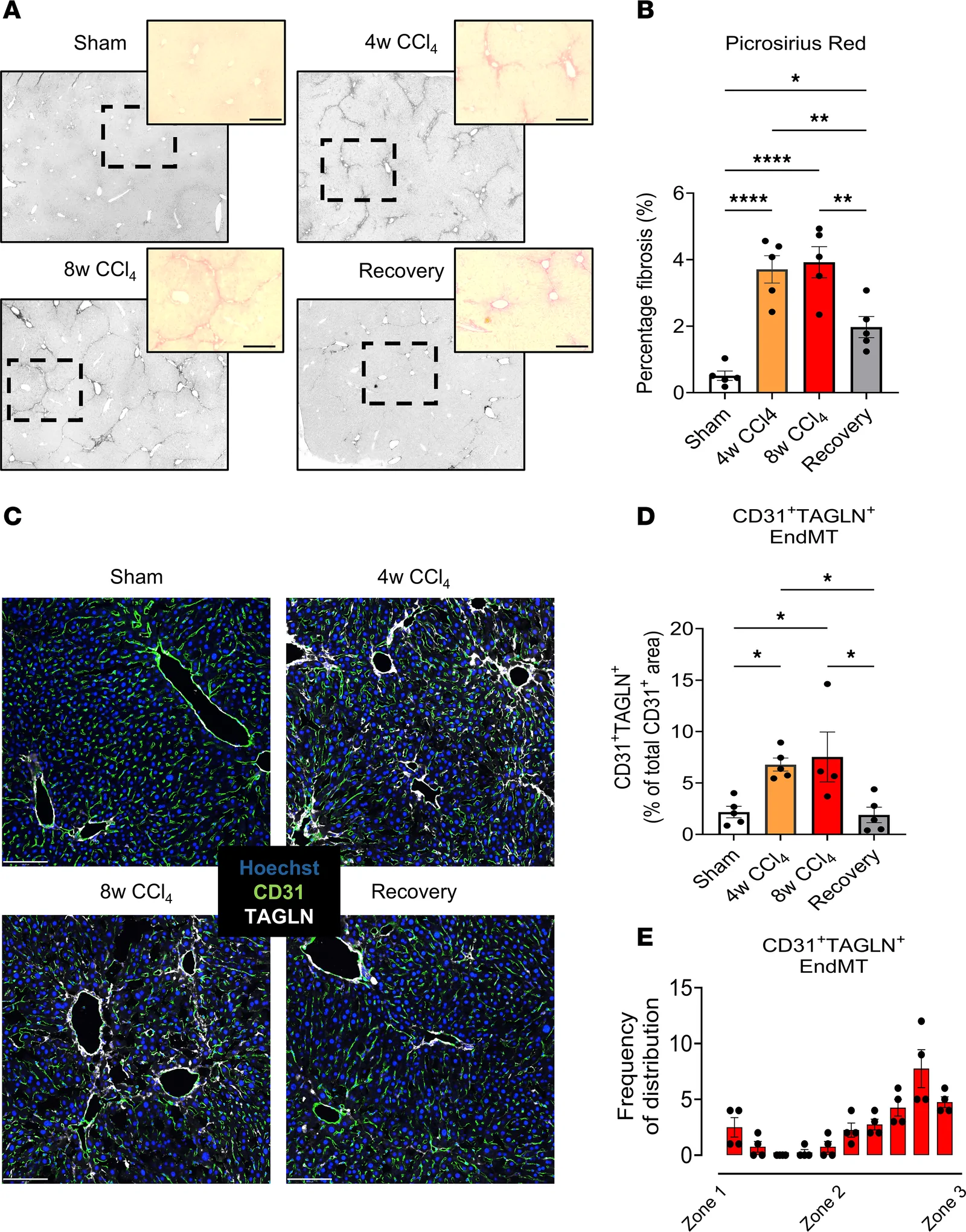
Induction of EndMT in response to CCl_4_-induced liver fibrosis and tissue recovery. (**A**) Representative images of picrosirius red staining of liver sections from Sham, 4-, and 8-week CCl_4_-treated and recovery groups. Scale bar: 250 μm. (**B**) Quantification of picrosirius red staining, expressed as a percentage (%) of total tissue area. (**C**) Representative confocal images of IF staining for Hoechst (blue), CD31 (green), and transgelin (TAGLN; white) in Sham, CCl_4_-treated, and recovery groups. Scale bar: 100 μm. (**D**) Quantification of CD31^+^TAGLN^+^ EndMT cells expressed as a percentage of the total CD31^+^ area. (**E**) Bar graph of EndMT cell distribution across the portal vein to central vein axis. For statistical analysis, 1-way ANOVA with Tukey’s multiple-comparison test were used. *n* = 5 animals/group. *P < 0.05, **P < 0.01, ****P < 0.0001.

**Figure 4:**
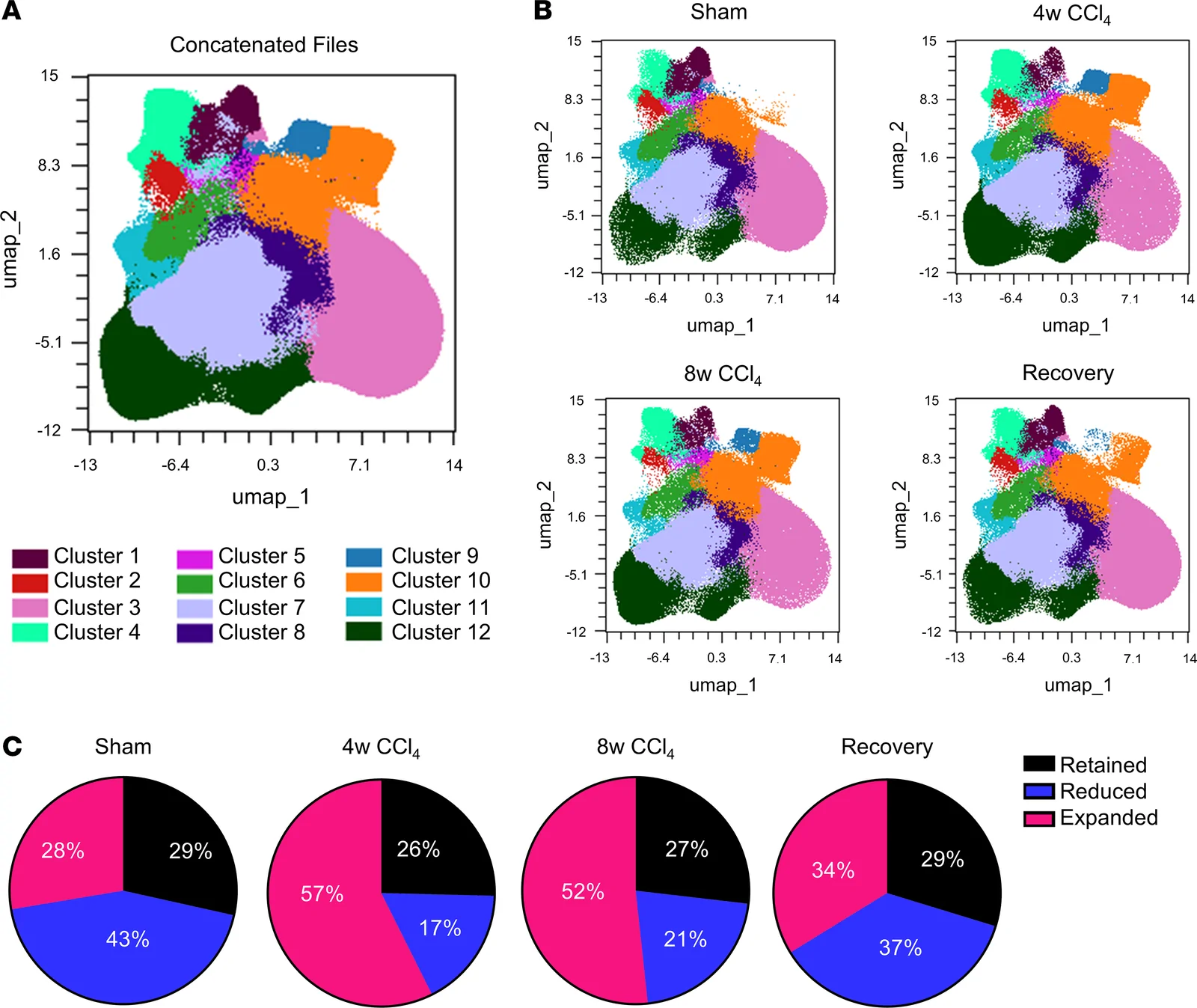
Mapping EC proteomic changes in response to CCl_4_-induced liver fibrosis and tissue recovery. (**A** and **B**) High-dimension reduction analysis displayed as UMAP of CD45^–^CD31^+^ cells from concatenated files for control, CCl_4_-treated, and recovery group samples displaying 12 distinct endothelial cell clusters and CD45^–^CD31^+^ cells split by treatment group. (**C**) Pie charts of the percentage composition of total EC characterized as Retained (black), Reduced (blue), and Expanded (pink) for each treatment group. *n* = 5 per group.

**Figure 5:**
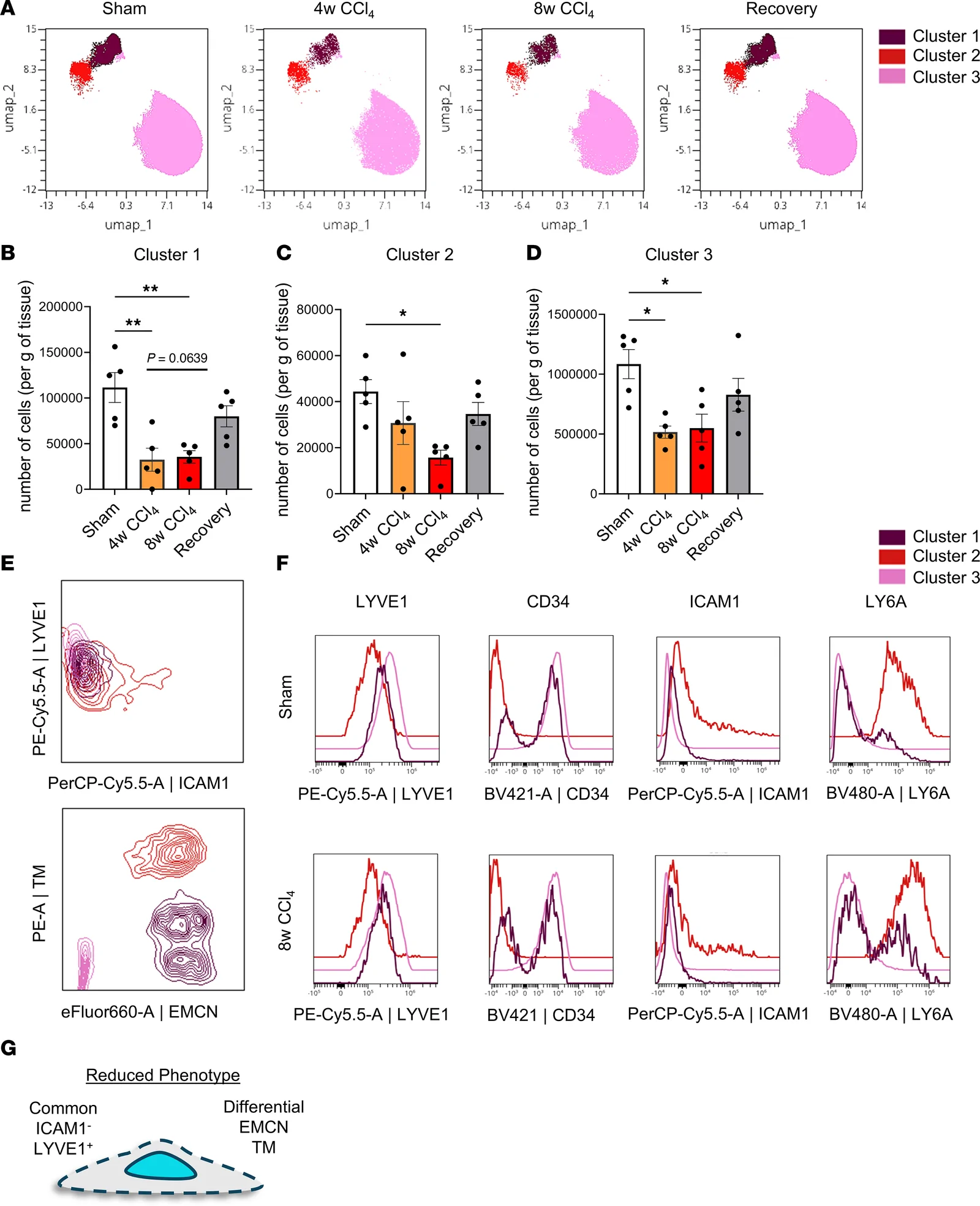
Phenotyping the characteristics of reduced EC subpopulations by FSFC. (**A**) UMAP visualization of 3 Reduced EC clusters; 1 (purple), 2 (red), and 3 (pink). (**B**–**D**) Quantification of the number of cells in each cluster expressed as number of cells per gram of liver tissue for Cluster 1 (**B**), Cluster 2 (**C**), and Cluster 3 (**D**) per treatment group. (**E**) Contour plots of clusters 1, 2, and 3 for ICAM1 versus LYVE1 (top panel) and endomucin (EMCN) versus thrombomodulin (TM) (bottom panel). (**F**) Histograms of clusters 1, 2 and 3 comparing the median fluorescence intensity (MFI) of LYVE1, CD34, ICAM1, and LY6A in Sham (top panels) and 8w CCl_4_–treated animals (bottom panels). (**G**) Schematic summarizing the common and differential traits of the 3 Reduced EC clusters. For statistical analysis, 1-way ANOVAs with Tukey’s multiple-comparison tests were used for **B** and **D** and a Kruskal-Wallis test with Dunn’s multiple-comparison test was used for **C**. *n* = 5 animals/group. *P < 0.05, **P < 0.01.

**Figure 6:**
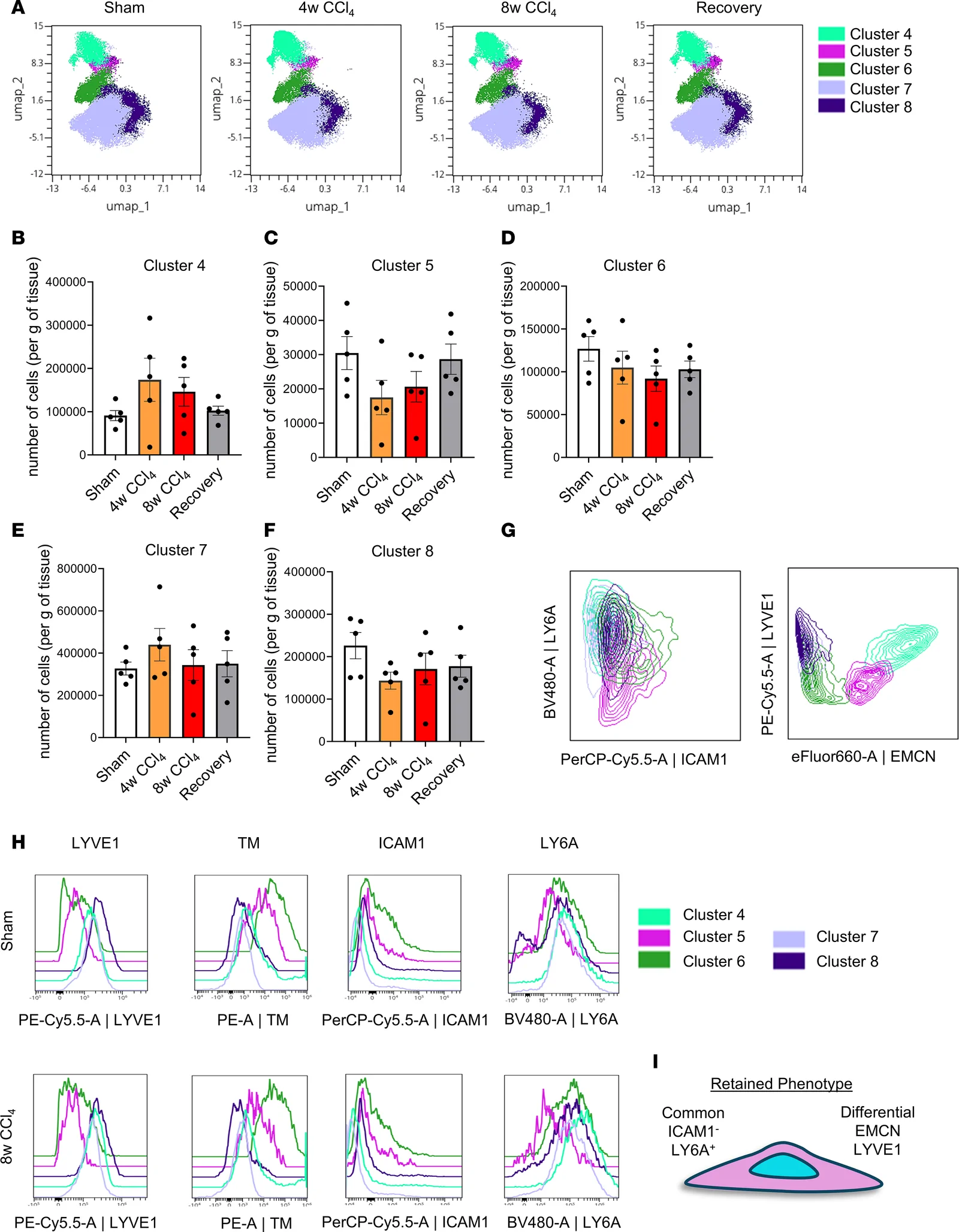
Phenotyping the characteristics of retained EC subpopulations by FSFC. (**A**) UMAP visualization of 5 Retained EC clusters; 4 (turquoise), 5 (light purple), 6 (green), 7 (violet), and 8 (dark purple). (**B**–**F**) Quantification of the number of cells in each cluster expressed as number of cells per gram of liver tissue for Cluster 4 (**B**), Cluster 5 (**C**), Cluster 6 (**D**), Cluster 7 (**E**), and Cluster 8 (**F**) per treatment group. (**G**) Contour plots of clusters 4, 5, 6, 7, and 8 for ICAM1 versus LY6A (top panel) and EMCN versus LYVE1 (bottom panel). (**H**) Histograms of clusters 4, 5, 6, 7, and 8 comparing the MFI of LYVE1, TM, ICAM1, and LY6A in Sham (top panels) and 8w CCl_4_–treated animals (bottom panels). (**I**) Schematic summarizing the common and differential traits of Retained EC clusters. For statistical analysis, 1-way ANOVAs with Tukey’s multiple-comparison tests. *n* = 5 animals/group.

**Figure 7:**
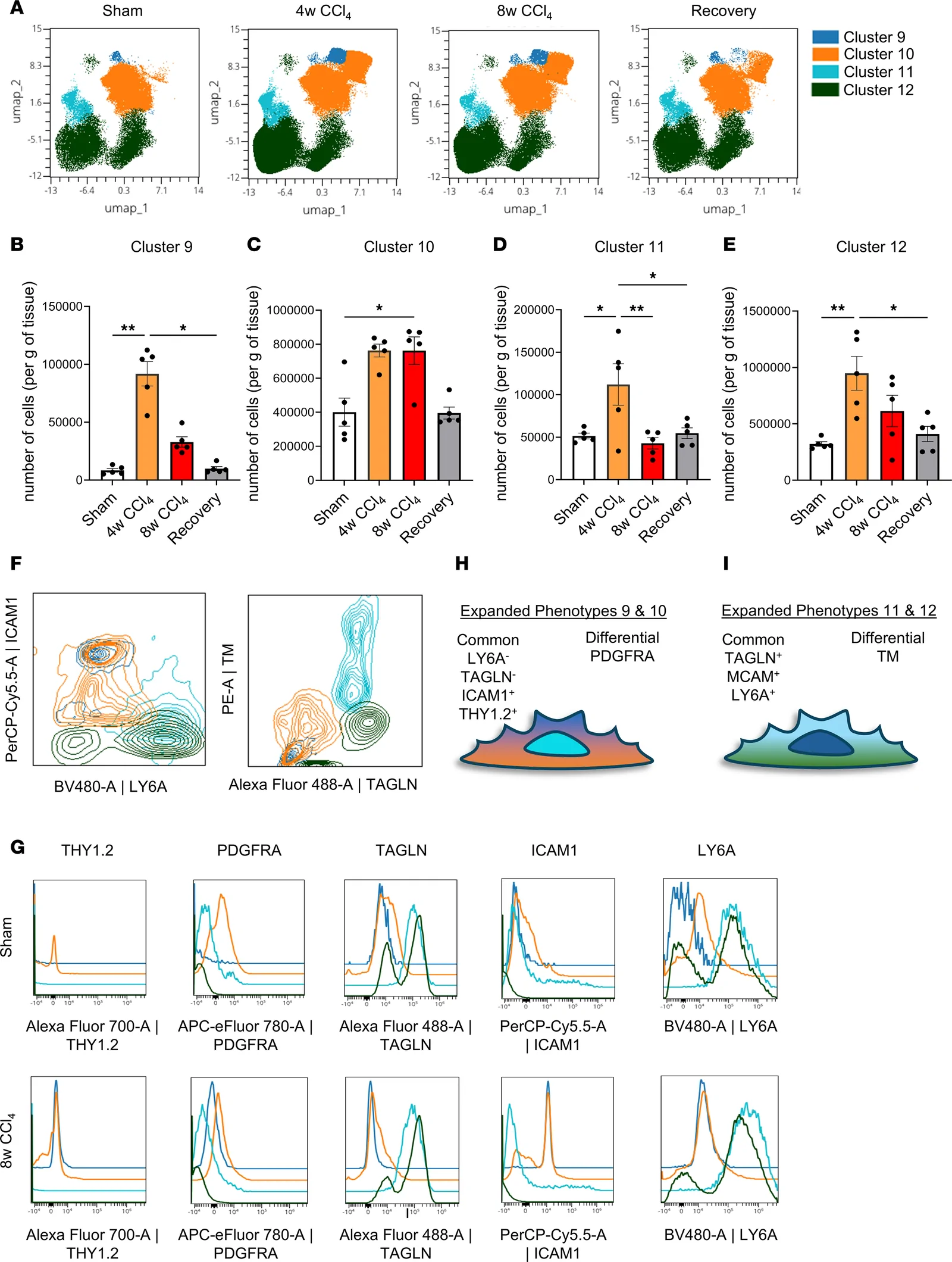
Phenotyping the characteristics of expanded EC clusters by FSFC. (**A**) UMAP visualization of 4 Expanded EC clusters; 9 (dark blue), 10 (orange), 11 (teal), and 12 (dark green). (**B**–**E**) Quantification of the number of cells in each cluster expressed as number of cells per gram of liver tissue for Cluster 9 (**B**), Cluster 10 (**C**), Cluster 11 (**D**), and Cluster 12 (**E**) per treatment group. (**F**) Contour plots of clusters 9, 10, 11, and 12 for LY6A versus ICAM1 (top panel) and transgelin (TAGLN) versus thrombomodulin (TM) (bottom panel). (**G**) Histograms of clusters 9, 10, 11, and 12 comparing the mean fluorescence intensity (MFI) for THY1.2, PDGFRα, TAGLN, ICAM1, and LY6A in Sham (top panels) and 8w CCl_4_–treated animals (bottom panels). (**H** and **I**) Schematics summarizing the common and differential traits of clusters 9 and 10 (**H**) as well as clusters 11 and 12 (**I**). For statistical analysis, Kruskal-Wallis tests with Dunn’s multiple-comparison test were used for **B** and **C** and 1-way ANOVA with Tukey’s multiple-comparison test were used for **D** and **E**. *P < 0.05, **P < 0.01.

**Figure 8:**
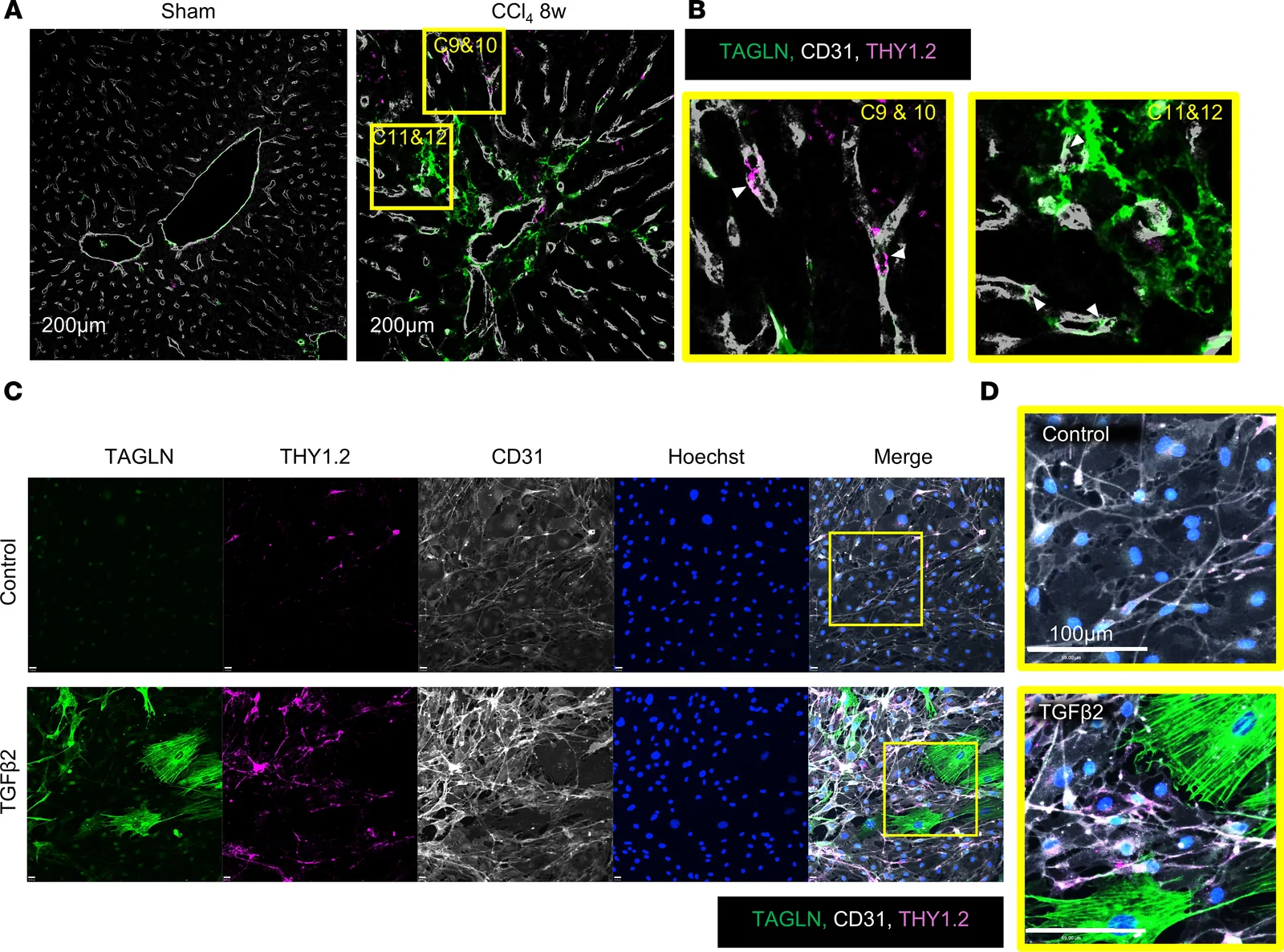
TAGLN^+^ and THY1.2^+^ expanded EC clusters display distinct localization and morphology. (**A**) Representative confocal images of IF staining for CD31 (white), THY1.2 (magenta), and transgelin (TAGLN; green) in Sham and 8w CCl_4_–treated liver sections. Scale bar: 200 μm. (**B**) Magnified regions with left panel identifying THY1.2^+^ FAEC (white arrowheads) and right panel identifying TAGLN^+^ FAEC (white arrowheads). (**C**) Representative confocal images of IF staining for CD31 (white), THY1.2 (magenta), TAGLN (green), and Hoechst (blue) from isolated mouse EC cultured in the presence of either media alone or 10 ng/mL TGF-β2 for 48h. Scale bar: 100 μm. (**D**) Magnified examples of THY1.2^+^ FAEC cells and TAGLN^+^ FAEC cells in control (left panel) and TGF-β2–treated cells (right panel). Scale bar: 100 μm.

**Figure 9:**
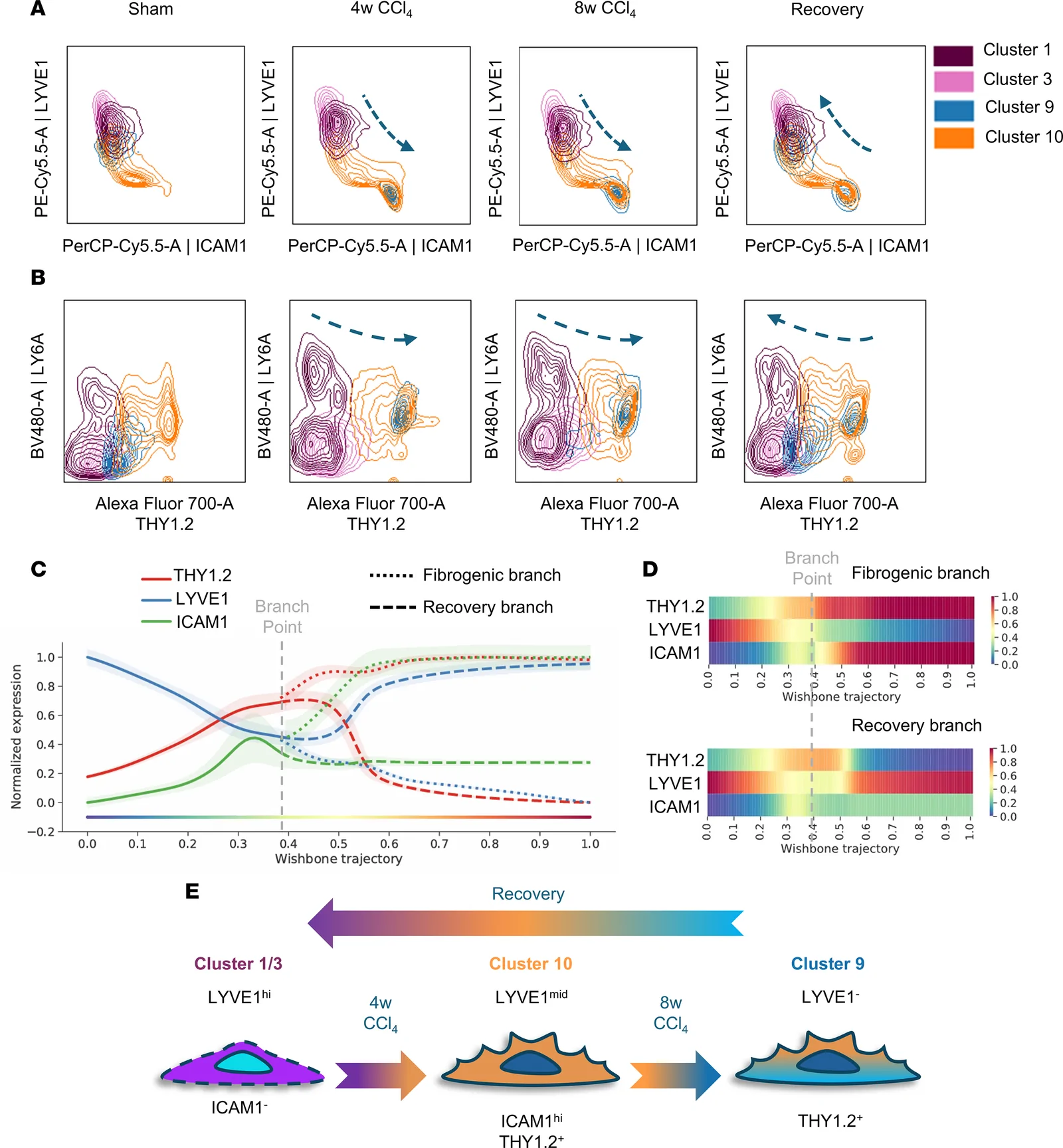
Derivation of THY1.2^+^ expanded FAEC from reduced EC clusters through acquisition of EndMT traits. (**A** and **B**) Contour plots of clusters 1, 3, 9, and 10 for ICAM1 versus LYVE1 and THY1.2 versus LY6A. Arrow denotes the shifting phenotypic profile of the clusters for the different treatment groups. (**C**) Wishbone trajectory analysis of clusters 1, 3, 9, and 10 for THY1.2 (red), LYVE1 (blue) and ICAM1 (green). (**D**) Heatmap displaying the expression profile of the Fibrogenic branch (top panel) or Recovery branch (bottom panel) for THY1.2, LYVE1, and ICAM1. (**E**) Schematic summarizing the trajectory and profile of clusters 1, 3, 9, and 10 during fibrogenesis and tissue recovery. *n* = 5 per group.

**Figure 10:**
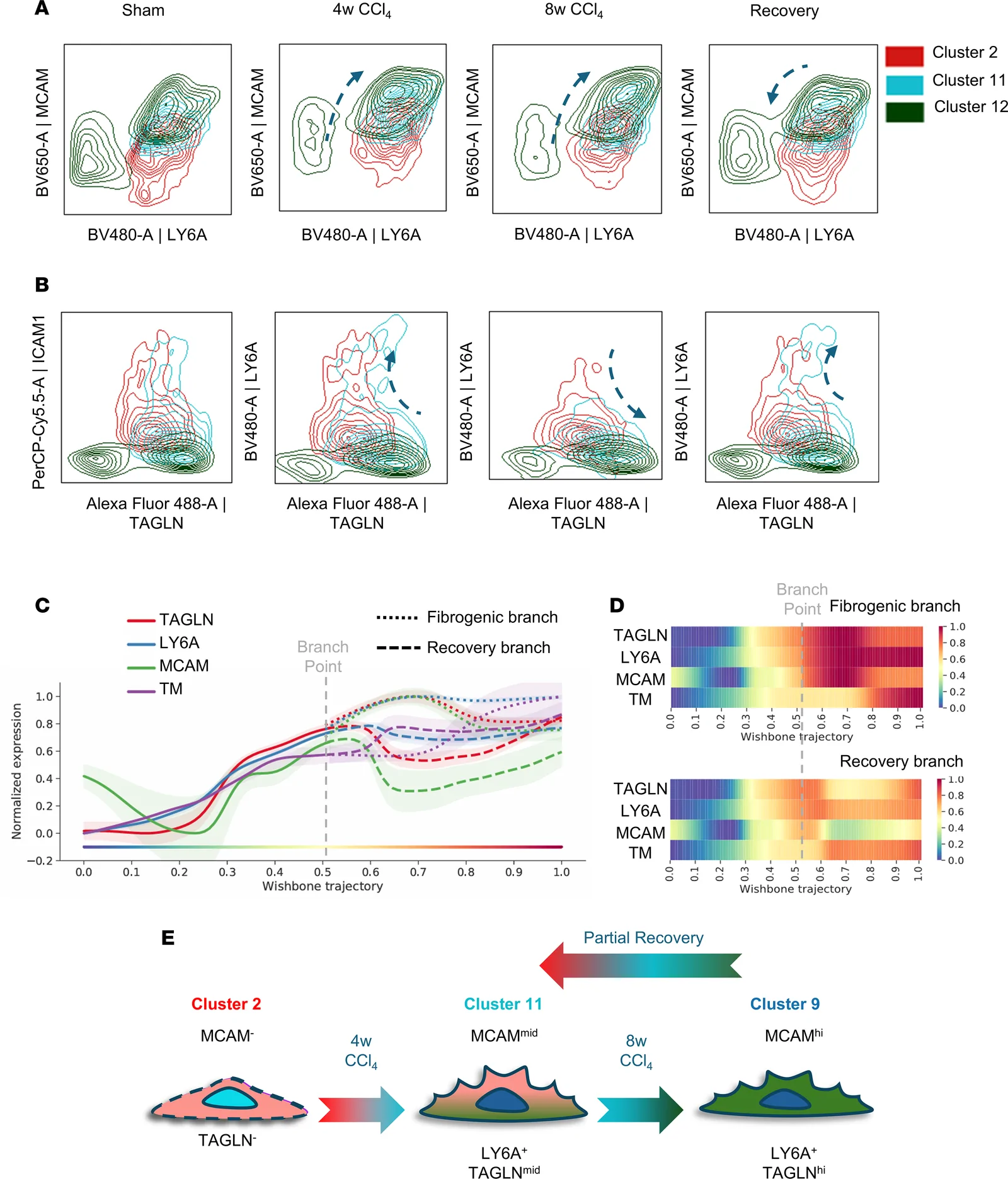
Derivation of TAGLN^+^ expanded FAEC from reduced EC clusters through acquisition of EndMT traits. (**A** and **B**) Contour plots of clusters 2, 11, and 12 for LY6A versus MCAM (**A**) and transgelin (TAGLN) versus ICAM1 (**B**). Arrow denotes the shifting phenotypic profile of the clusters for the different treatment groups. (**C**) Wishbone trajectory analysis of clusters 2, 11, and 12 for TAGLN (red), LY6A (blue), MCAM (green), and TM (purple). (**D**) Heatmap displaying the expression profile of the Fibrogenic branch (top panel) or Recovery branch (bottom panel) for TAGLN, LY6A, MCAM, and thrombomodulin (TM). (**E**) Schematic summarizing the trajectory and profile of clusters 2, 11, and 12 during fibrogenesis and tissue recovery. *n* = 5 per group.

**Figure 11:**
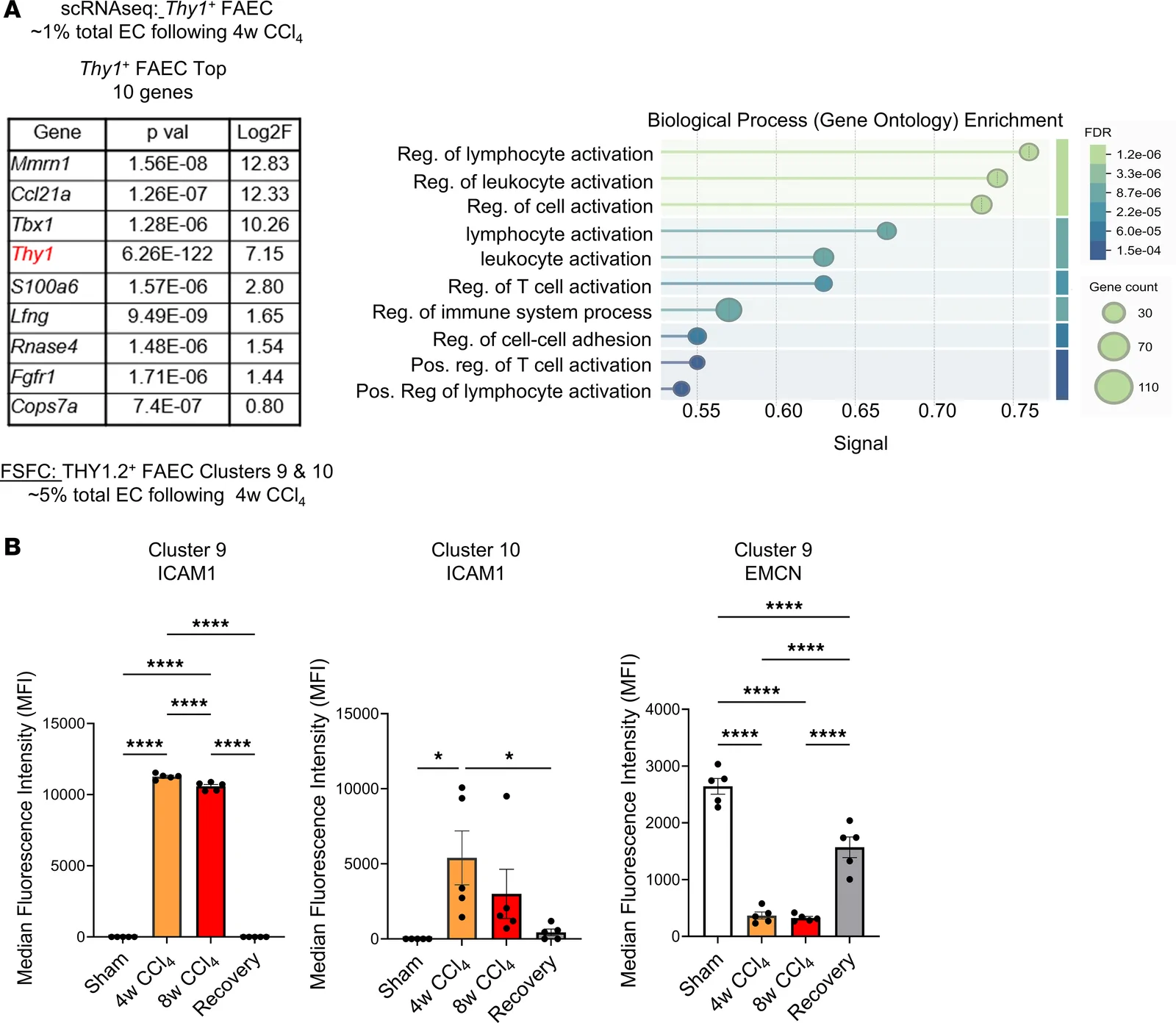
Comparative analysis of scRNA-seq *Thy1^+^* FAEC with FSFC profile of THY1.2^+^ FAEC. (**A**) ScRNA-seq analysis of *Thy1*-expressing liver EC from 4w CCl_4_–treated mice compared with all other EC. Table of the top 10 enriched genes and gene ontology analysis of putative biological processes enriched in *Thy1*^+^ EC based on their differentially expressed genes. (**B**) FSFC analysis of median fluorescence intensity (MFI) of THY1.2^+^ FAEC for ICAM1 and EMCN in Cluster 9 and Cluster 10. For statistical analysis, 1-way ANOVA with Tukey’s multiple-comparison tests were used. *n* = 5 animals/group. *P < 0.05, ****P < 0.0001.

**Figure 12:**
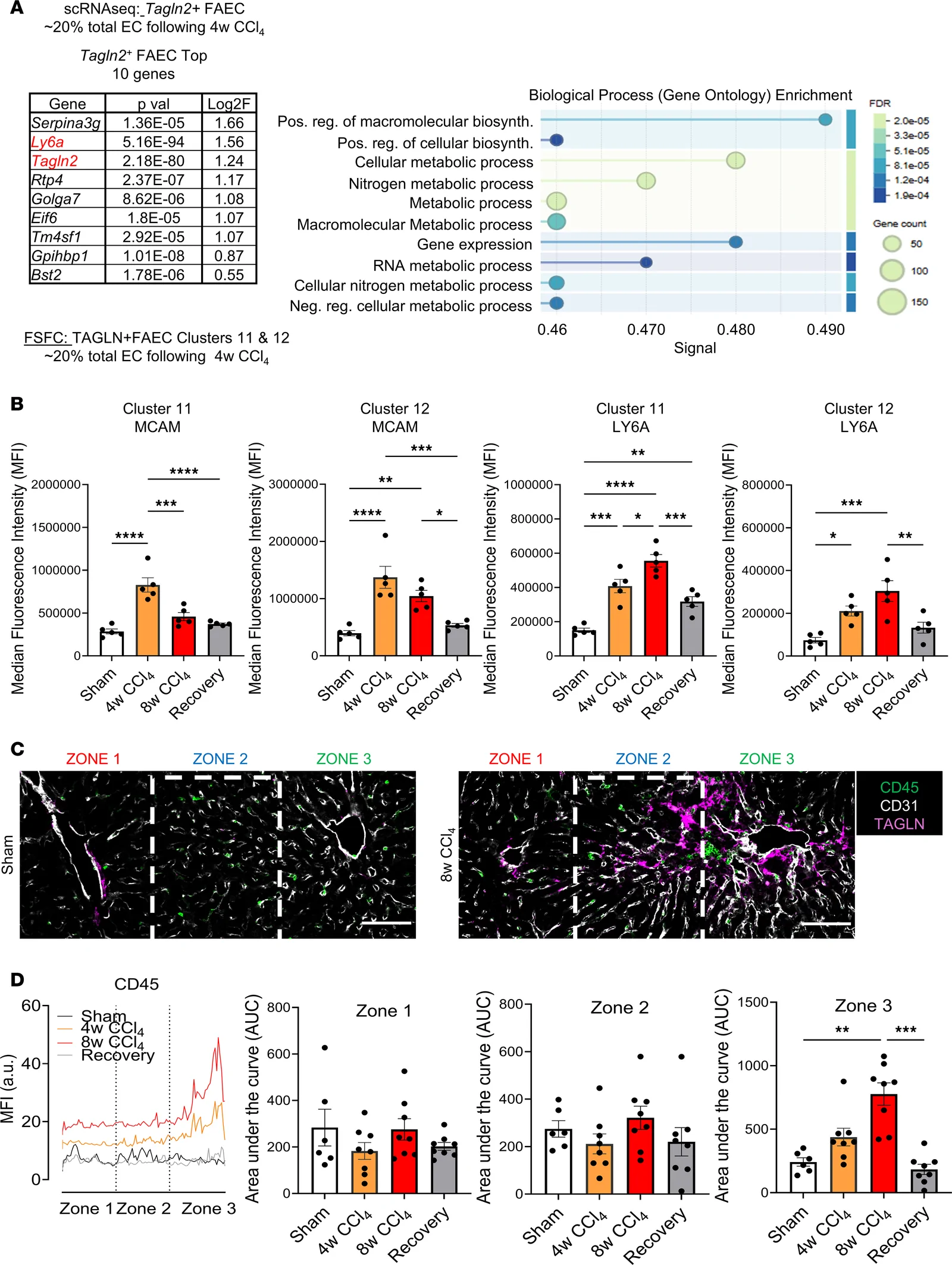
Comparative analysis of scRNA-seq *Tagln2^+^* FAEC with FSFC profile of TAGLN^+^ FAEC. (**A**) ScRNA-seq analysis of *Tagln2*-expressing liver EC from 4w CCl_4_–treated mice compared with all other EC. Table of the top 10 enriched genes and gene ontology analysis of putative biological processes enriched in *Tagln2^+^* EC based on their differentially expressed genes. (**B**) FSFC analysis of median fluorescence intensity (MFI) of TAGLN^+^ FAEC for MCAM and LY6A in Cluster 11 and 12. (**C**) Representative confocal images of IF staining for CD45 (green), CD31 (white), and TAGLN (magenta) in Sham and 8w CCl_4_–treated livers. Scale bar: 100 μm. (**D**) Profile plot of CD45^+^ MFI across the portal-central vein axis in Sham, CCl_4_-treated, and recovery experimental groups. For statistical analysis, 1-way ANOVA with Tukey’s multiple-comparison tests were used. *n* = 2 fields/animal per group. *P < 0.05, **P < 0.01, ***P < 0.001, ****P < 0.0001.

**Figure 13:**
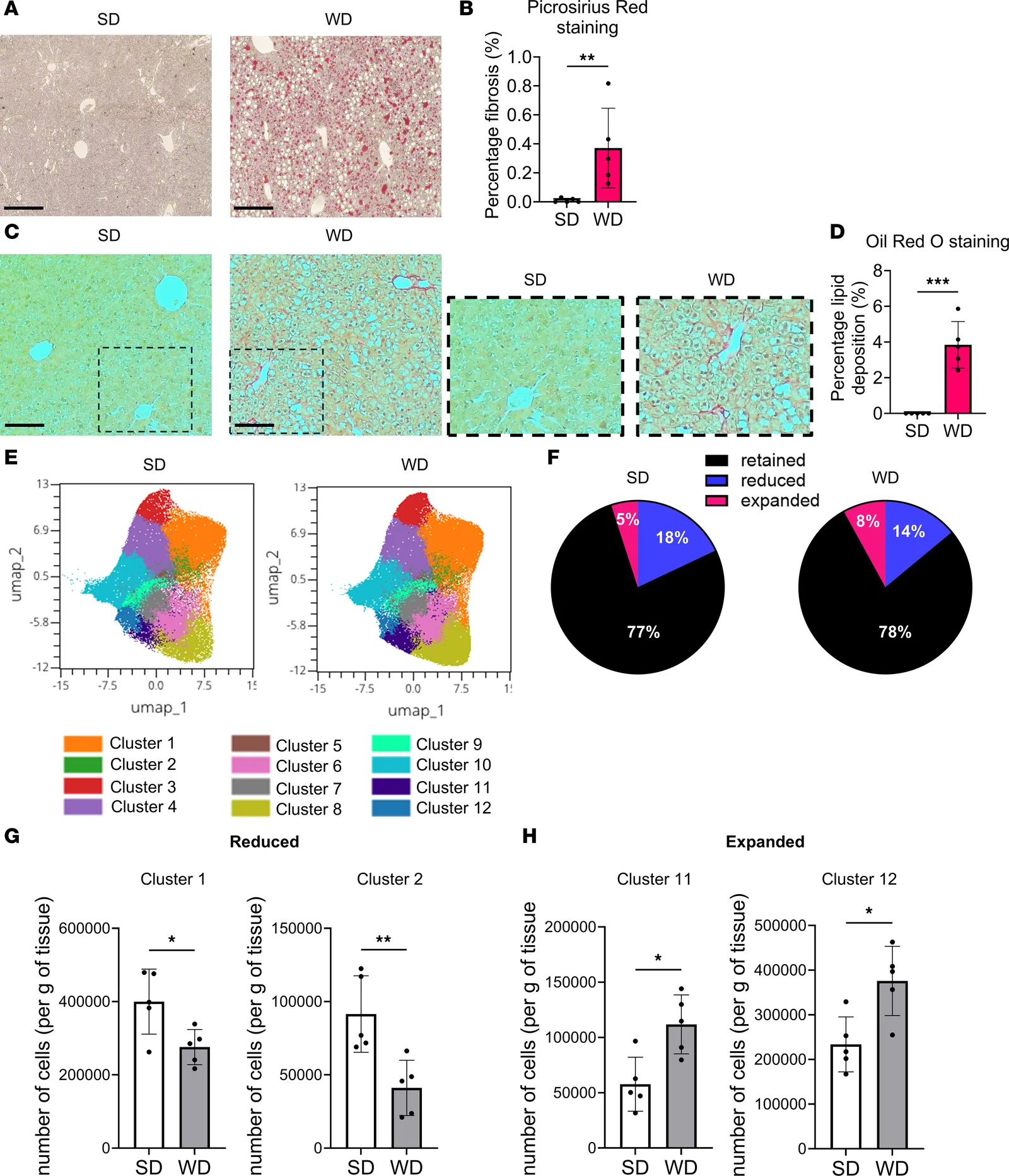
Expansion of TAGLN^+^ FAEC in a 12w Western diet model of MASLD. (**A**) Representative images of Oil Red O staining of liver samples from 12w standard diet-fed (SD-fed) and Western diet–fed (WD-fed) mice. Scale bars: 250 μm. (**B**) Quantification of Oil Red O staining expressed as a percentage (%) of lipids detected over the total tissue area. (**C**) Representative images of Picrosirius Red staining of liver samples from 12w SD- and WD-fed mice with zoomed in region. Scale bars: 250 μm. (**D**) Quantification of Picrosirius Red staining expressed as a percentage (%) of collagen detected over the total tissue area. (**E**) UMAP plots of CD45^–^CD31^+^ EC clusters from SD or WD fed mice. (**F**) Pie charts of the percentage composition of total EC characterized as Retained (black), Reduced (blue), and Expanded (pink) for each diet group. (**G** and **H**) Quantification of the number of cells per gram of liver tissue for Reduced Cluster 1 and Cluster 2 (**G**) and for Expanded Cluster 11 and Cluster 12 (**H**). For statistical analysis, unpaired *t* tests were used for **B**, **G**, and **H**, and a Mann-Whitney *U* test was used for **D**. *n* = 5 animals/group. *P < 0.05, **P < 0.01, ***P < 0.001.

**Figure 14:**
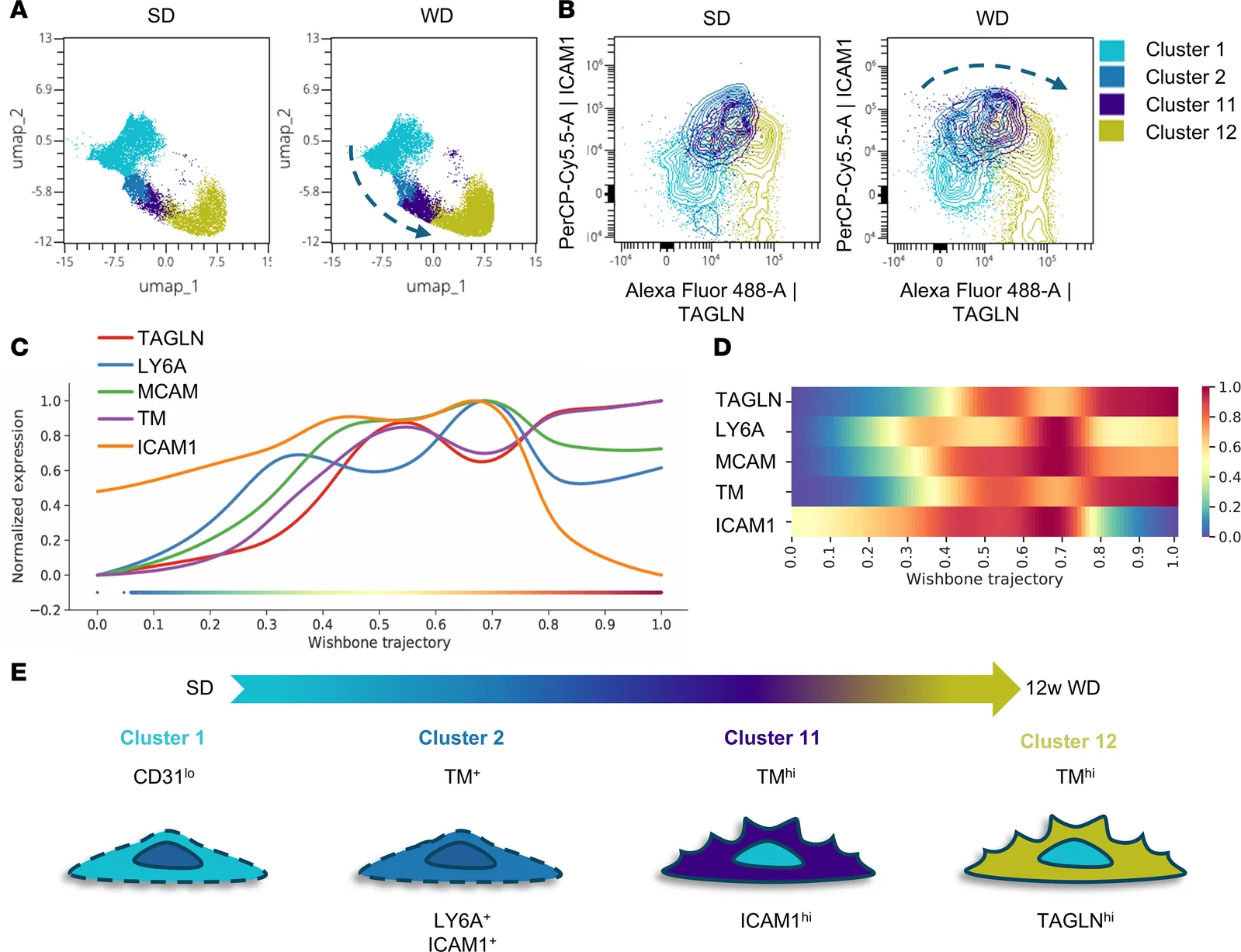
Derivation of TAGLN^+^ FAEC in a 12w Western diet model by Wishbone analysis. (**A**) UMAP of Cluster 1 (turquoise), Cluster 2 (blue), Cluster 11 (purple), and Cluster 12 (yellow) from standard diet (SD) or Western diet (WD) groups. (**B**) Contour plots of transgelin (TAGLN) versus ICAM1 for clusters 1, 2, 11, and 12 for SD or WD groups. Arrow denotes the shifting phenotypic profile of the clusters from SD to WD. (**C**) Wishbone trajectory analysis of clusters 1, 2, 11, and 12 for TAGLN (red), LY6A (blue), MCAM (green), TM (purple), and ICAM1 (orange). (**D**) Heatmap of the expression profile of each marker. (**E**) Schematic summary of the trajectory and profile of clusters 1, 2, 11, and 12.

**Figure 15:**
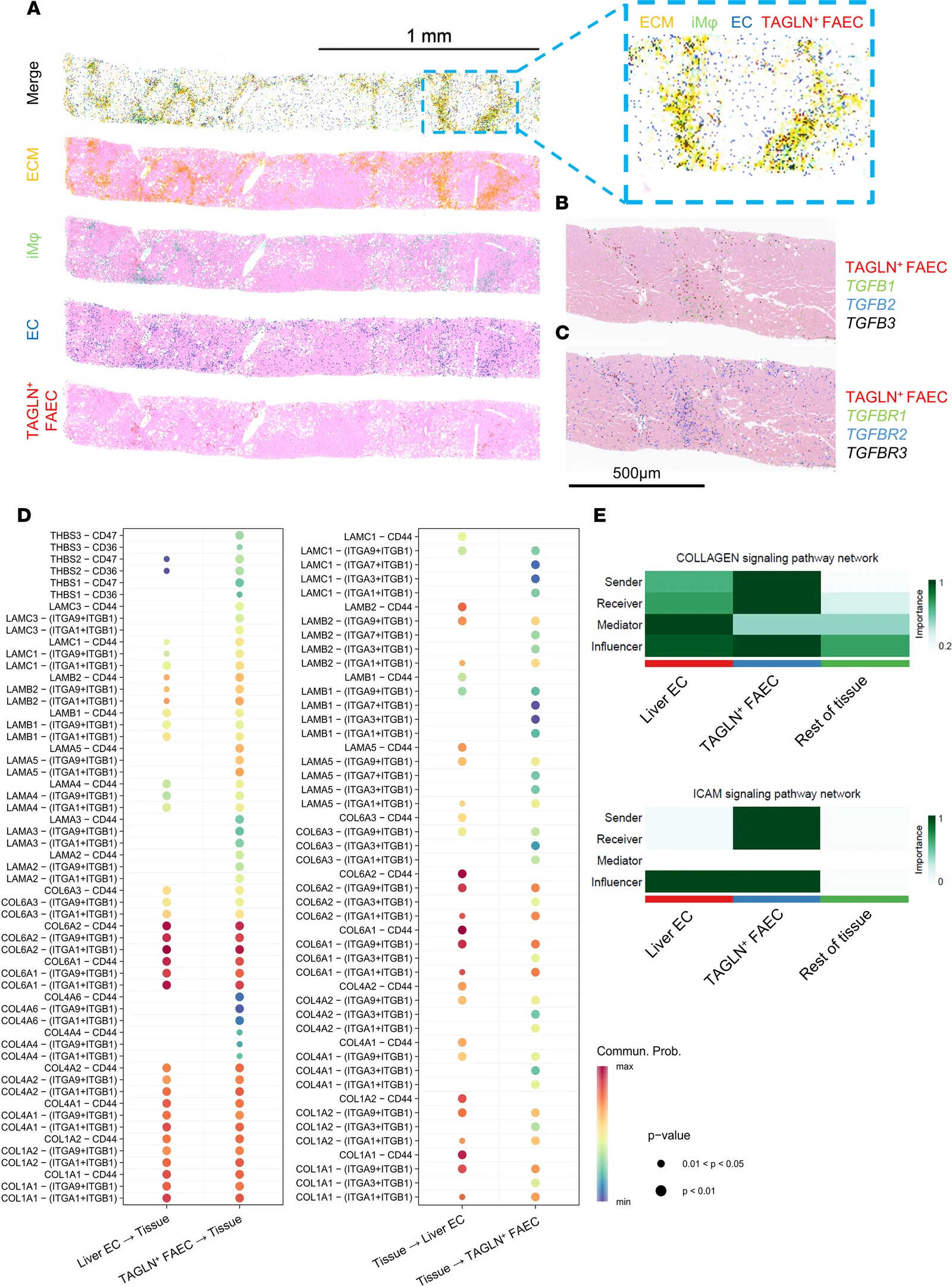
Spatial transcriptomics reveals TAGLN^+^ FAEC is a conserved feature of MASLD. (**A**) Visualization of spatial transcriptomic clusters for *k*-means shows enrichment data for a patient with Ishak score of 2–3 identifying extracellular matrix (ECM; yellow), inflammatory macrophages (iMϕ), all endothelial cells (EC; blue), and TAGLN^+^FAEC (red). Zoomed-in region highlighted in blue box. (**B** and **C**) Representative images of the colocalization of TAGLN^+^ FAEC (red) with TGF-β isoforms (**B**: *TGFB1*, green; *TGFB2*, blue; *TGFB3*, black) and TGF-β family receptors (**C**: *TGFBR1*, green; *TGFBR2*, blue; *TGFBR3*, black). (**D**) CellChat analysis comparing enriched of putative interaction patterns from EC to tissue and tissue to EC. (**E**) Comparison between all Liver EC and TAGLN^+^ FAEC for collagen and ICAM signaling network displayed as heatmaps.

**Figure 16:**
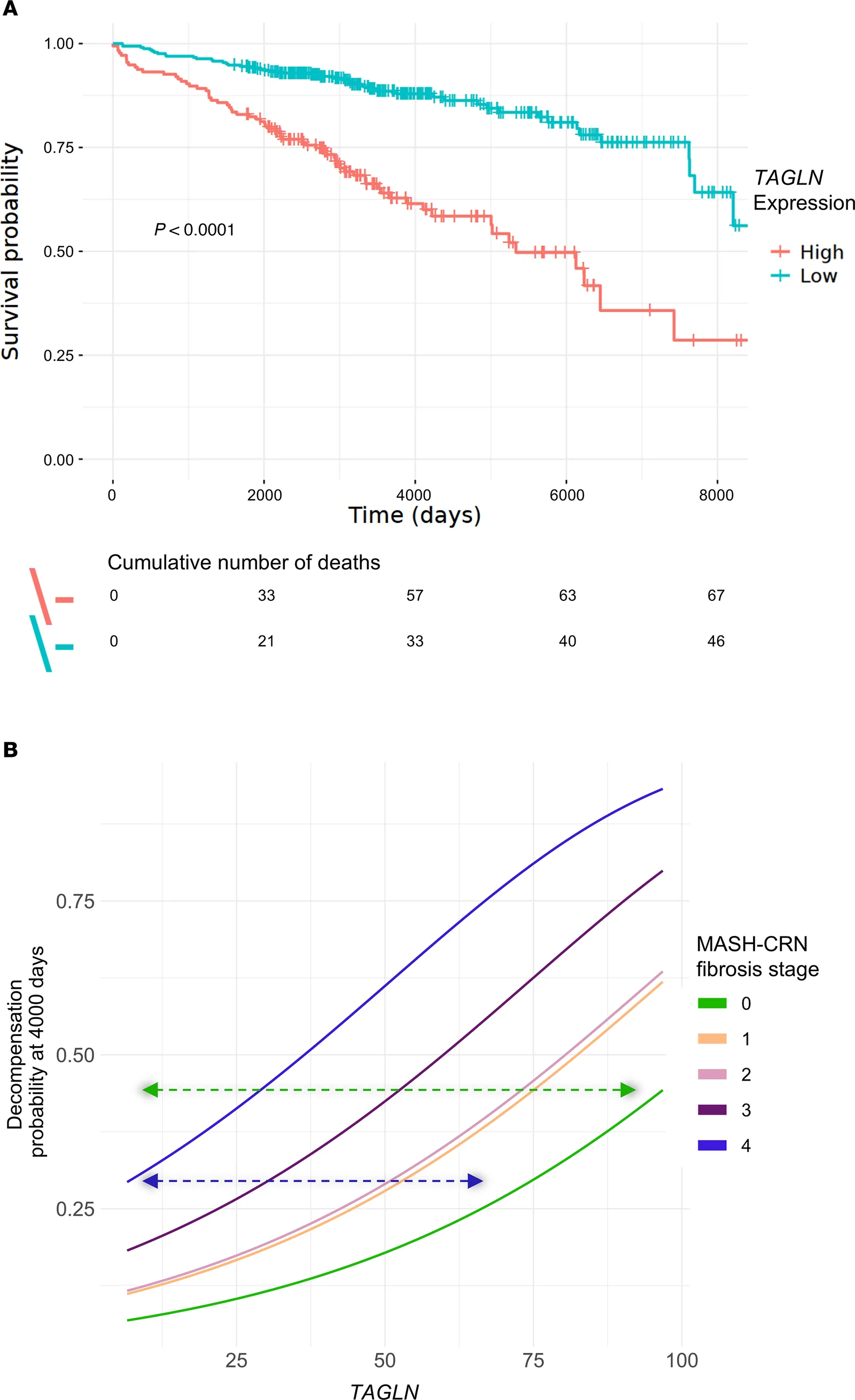
TAGLN^+^ FAEC are a putative biomarker of progressive disease. (**A**) Kaplan-Meier survival curves comparing patients stratified into high and low *TAGLN* expression groups (*P*-value of log-rank test comparing groups) in the biopsy subset of the SteatoSITE cohort (*n* = 505). (**B**) Cumulative incidence functions for liver-related events at 4,000 days after biopsy as a function of *TAGLN* expression, stratified by pathologist-scored MASH-CRN fibrosis stage (*n* = 435; F0 (green), F1 (orange), F2 (pink), F3 (purple), and F4 (blue). Scale bar: 50 μm. For statistical analysis, a 1-way ANOVA with Dunnett’s multiple-comparison test was used. **P < 0.01.

## Version 1 09/01/2026

In-Press Preview

## Version 2 10/08/2026

Electronic publication
