# Supplementary material for: Mapping vascular plasticity during fibrogenesis identifies fibrosis-associated endothelial cells in early-stage liver disease
Source: JCI Insight. 2026 Sep 1;11(19):e207327. doi: 10.1172/jci.insight.207327 (PMC13726414; doi:10.1172/jci.insight.207327)
Supplement: Supplementary file 1 [file jciinsight-11-207327-s172.pdf]

Supplementary Figure 1

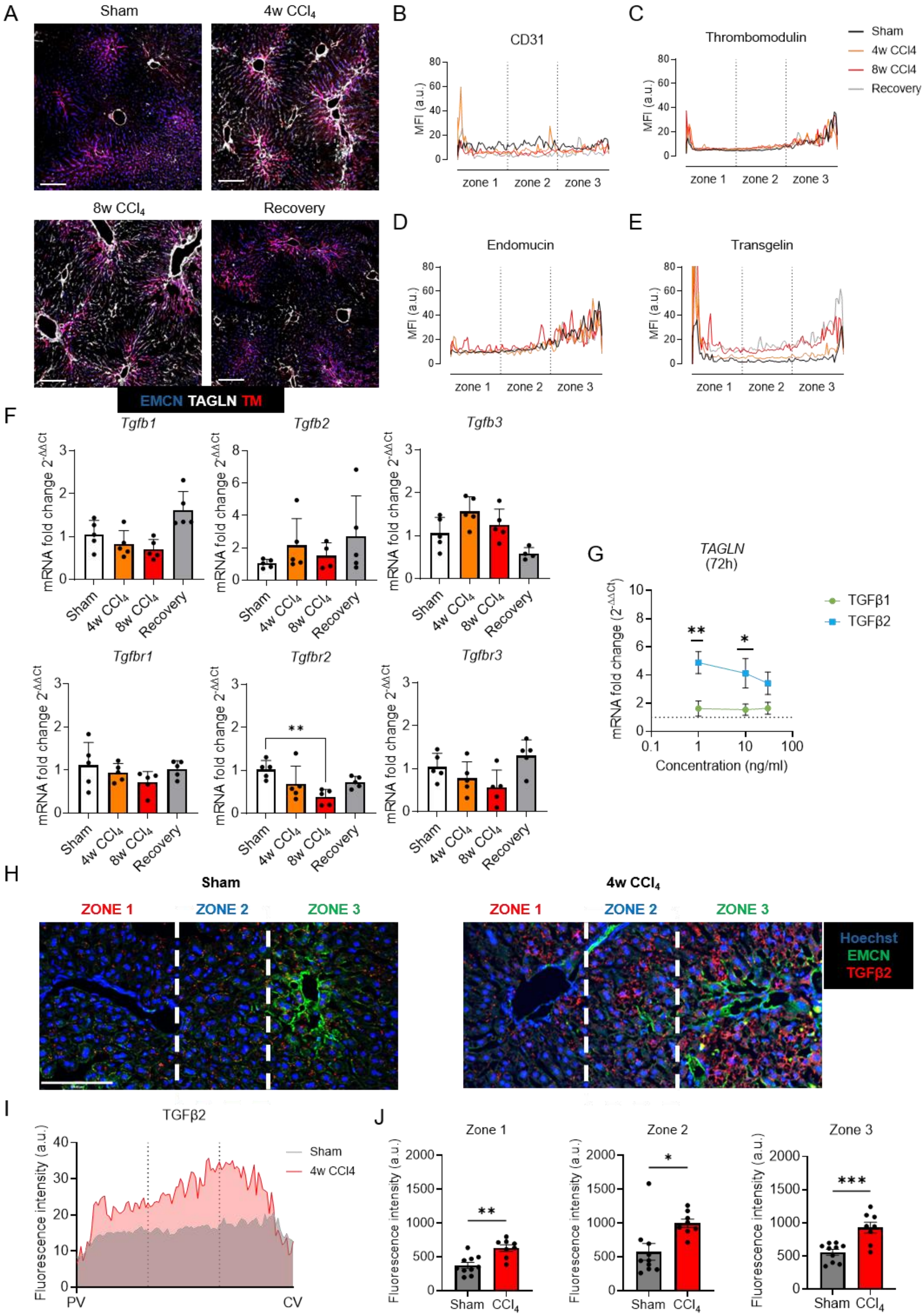

Supplementary Fig. 1. Endothelial plasticity and tissue remodelling in response to CCl_4-induced liver fibrosis. (A) Representative confocal images of thrombomodulin (TM) (red), endomucin (EMCN) (blue) and transgelin (TAGLN) (white) staining in the liver of Sham, 4w and 8w CCl_4-treated and Recovery group. Scale bar= 200μm. Profile plots for (B) CD31, (C) TM, (D) EMCN and (E) TAGLN showing the shift in zonation in Sham (black), 4w CCl_4 (orange), 8w CCl_4 (red) and recovery (grey) groups. (F) RT-PCR analysis of each TGFβ isoform and receptor family members. (G) Dose response curves for induction of TAGLN gene expression by TGFβ1 and TGFβ2 stimulation of HUVEC. HUVEC were treated with 1, 10 or 30ng/ml of TGFβ1 or TGFβ2 for 72 hours. N= 3 technical replicates/condition. For statistical analysis, a two-way ANOVA with Tukey's multiple comparisons was used. (H) Representative confocal images of nuclei (blue), EMCN (green) and TGFβ2 (red) for SD (left panel) and WD (right panel). Scale bar= 100μm. (I) Profile plots for TGFβ2 comparing SD with WD (J) with quantification of expression changes for each zone. (F) *; $p<0.05$, **; $p<0.01$, ***; $p<0.001$. N=5 per group, (J) N=10 (2 fields/animal per group)

Supplementary Figure 2

A

Target	Markers
Live Dead	Viability
CD45	Pan-Leukocyte

Target	Markers	Target	Markers
CD31	EC Identity	LY6A	Activation
TM	EC Identity	ICAM1	Activation
EMCN	EC Identity	CD34	Activation
MCAM	EC Identity	TAGLN	Mesenchymal
LYVE1	EC Identity	PDGFRα	Mesenchymal
PDPN	EC Identity	THY1.2	Mesenchymal

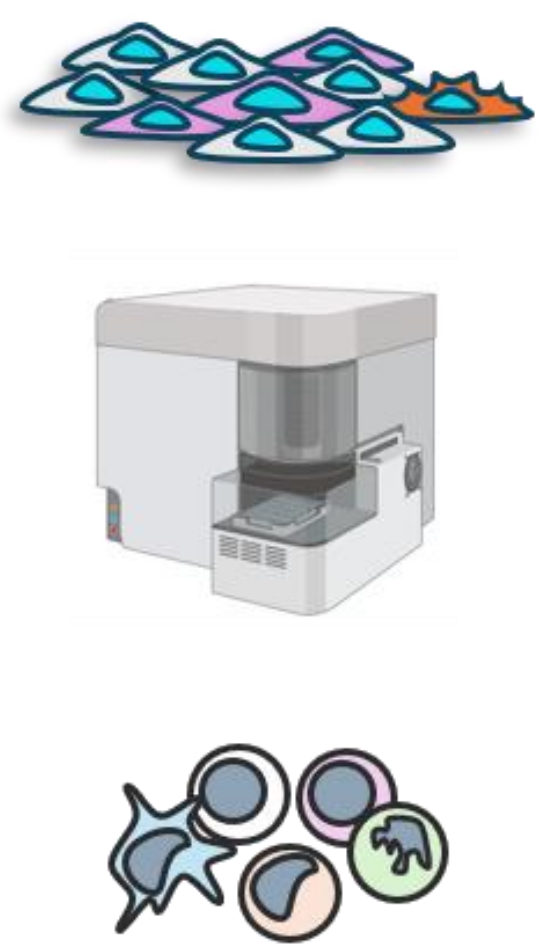

Target	Markers
Live Dead	Viability
CD31	EC

Target	Markers	Target	Markers
CD45	Pan-Leukocyte	CD3	T cell
CD11b	Myeloid/Granulocyte	CD4	T cell
LY6G	Granulocyte	CD8a	T cell
LY6C	Monocyte	TCRβ	T cell
F4/80	Mφ	CD19	B cell
CD64	Mφ	B220	B cell
CX3CR1	Mφ	XCR1	DC
TIM4	KC	CD11c	DC
NK1.1	NK cell	MHCII	APC

B

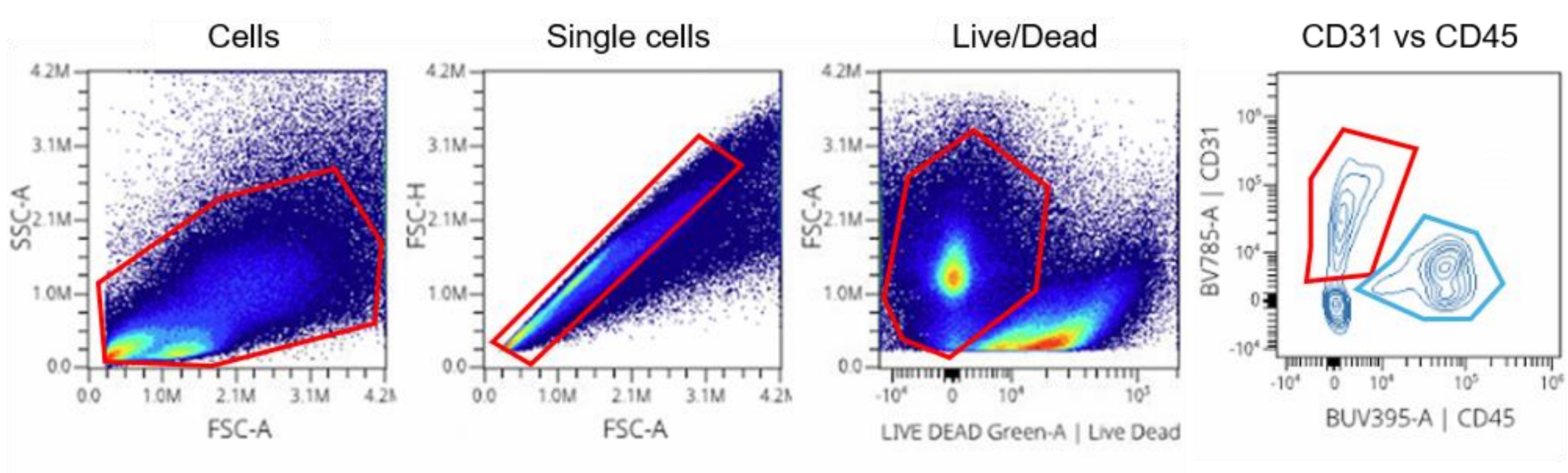

Supplementary Fig. 2. FSFC EC panel and gating. (A) Schematic for endothelial (blue) and immune cell (green) FSFC panels and (B) Gating strategy for the analysis of either CD45$^-$CD31$^+$ endothelial cells or CD45$^+$CD31$^-$ leukocytes. From left to right: a gate for all cells was firstly drawn using the forward scatter area (FSC-A) vs the side scatter area (SSC-A) plot. Then, single cells were selected using the FSC-A vs forward scatter height (FSC-H) plot. The dead cells were excluded from the analysis using the Live/Dead Fixable stain vs FSC-A plot. In the live cells gate, the CD45$^-$CD31$^+$ and CD45$^+$CD31$^-$ cells were gated

separately using the CD45 vs CD31 plot. The $CD45^{-}CD31^{+}$ gate (EC; red) and $CD45^{+}CD31^{-}$ (immune cells; blue).

Supplementary Figure 3

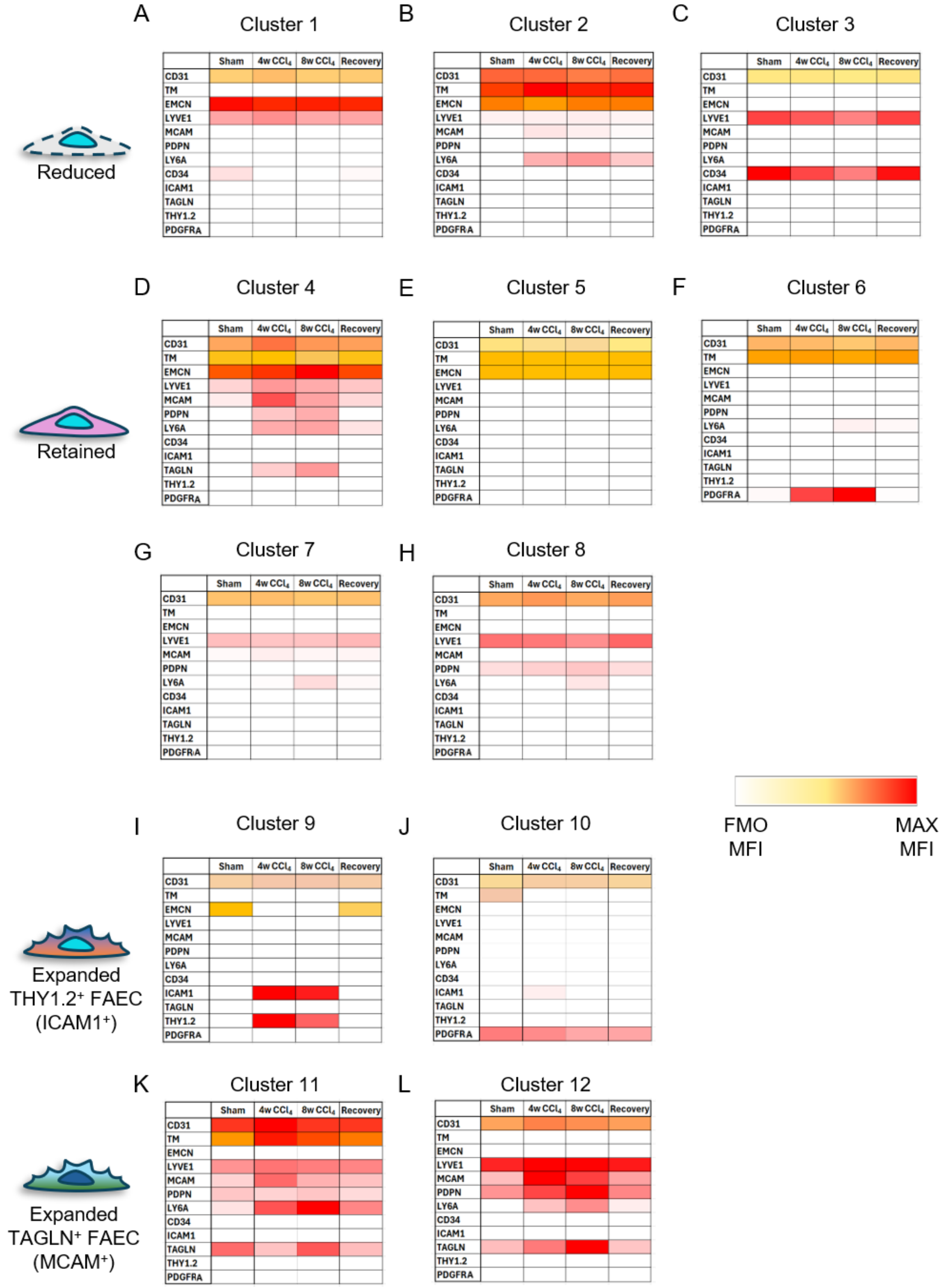

Supplementary Fig. 3. Heatmaps of the phenotypic properties of each EC cluster split by treatment group. Heatmaps of median fluorescence intensity (MFI) for (A-C) Reduced clusters 1-3 (D-H) Retained clusters 4-8 and (I-L) Expanded clusters 9-12. The heatmaps were created using full spectrum flow cytometry data. The fluorescence minus one (FMO) MFI for each marker was used as the threshold for positive signal. N=5 per group.

Supplementary Figure 4

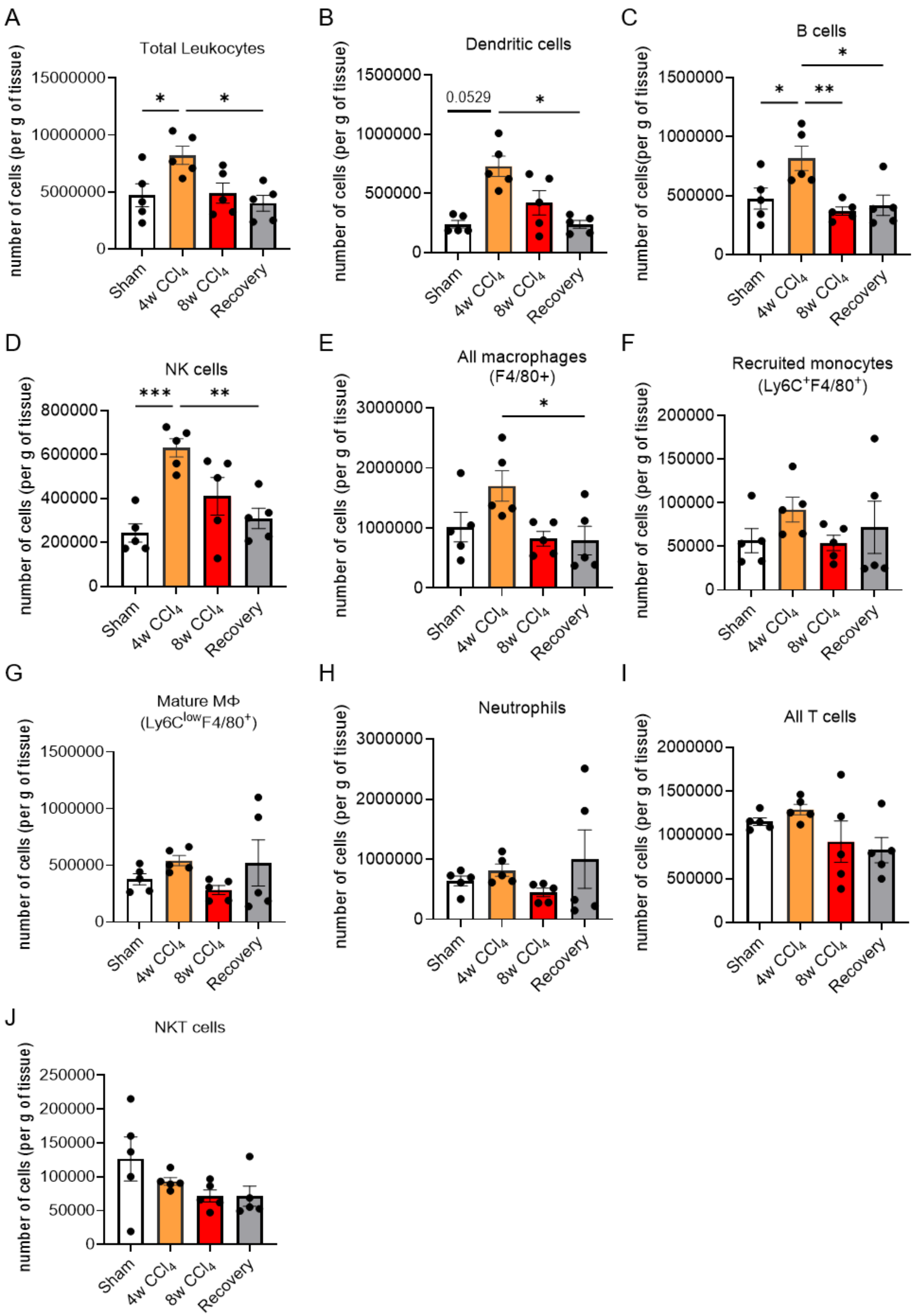

Supplementary Figure 4. Immune cell full spectrum flow cytometry profile in response to CCl_4 and tissue recovery. Quantification of (A) total number of $CD45^+$ leukocytes, (B) dendritic cells, (C) B cells, (D) NK cells, (E) macrophages, (F) recruited monocytes, (F) mature macrophages, (G) Mature macrophages, (H) neutrophils, (I) T cells and (J) NKT cells. For statistical analysis, one-way ANOVAs with Tukey's multiple comparisons tests were used, except for Figure B, where a Kruskal-Wallis test Dunn's multiple comparisons was used. *; $p<0.05$, **; $p<0.01$, ***; $p<0.001$. N= 5 animals/group.

Supplementary Figure 5

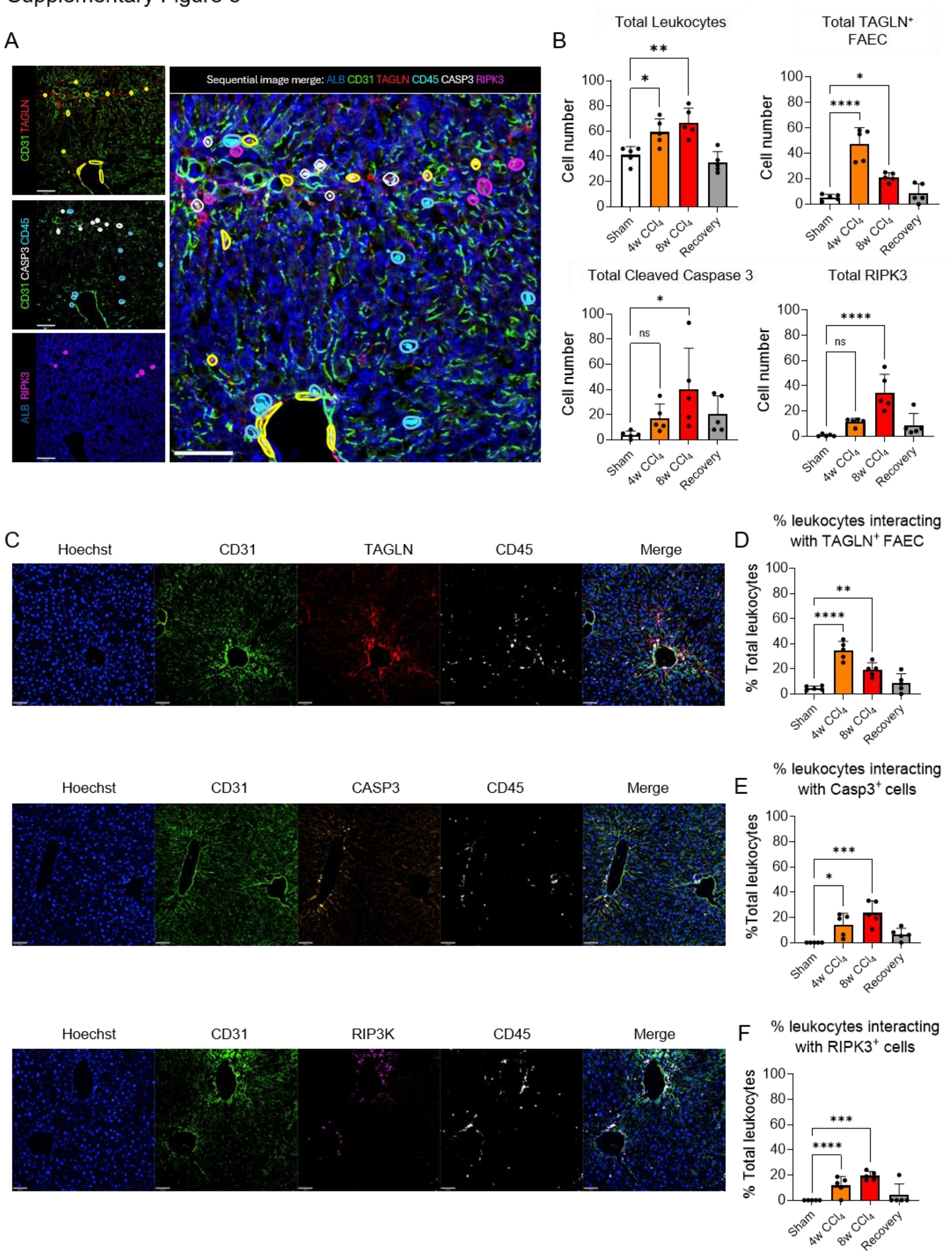

Supplementary Figure 5. Leukocytes interaction with TAGLN$^+$ FAEC, apoptotic and necrotic cells in response to CCl_4 and tissue recovery. (A) Representative confocal IF images showing individual panels for CD31 (green) and TAGLN (red), cleaved caspase 3 (CASP3; white), CD45$^+$ (light blue), albumin (ALB; dark blue), and receptor-interacting serine/threonine-protein kinase 3 (RIPK3; magenta). Overlay displays outlines for TAGLN$^+$ FAEC (yellow), CASP3$^+$ (white) and cells outlined to highlight localisation after 4-week CCl_4 treatment. Scale bars= 200µm. (B) Quantification of total CD45$^+$, TAGLN$^+$ FAEC, CASP3$^+$ and RIPK3$^+$ objects counted per field for Sham, 4w CCl_4, 8w CCl_4 and recovery groups. (C) Top to bottom shows representative split images of TAGLN (red), CASP3 (orange) and RIPK3 (magenta) with nuclei (blue), CD31 (green) and CD45 (white). Quantification of the percentage total CD45$^+$ leukocytes which are touching (D) TAGLN$^+$FAEC, (E) Casp3$^+$ and (F) RIPK3$^+$ objects per each animal. For statistical analysis one-way ANOVA tests with Tukey's multiple comparisons test were used. *; $p<0.05$, **; $p<0.01$, ***; $p<0.001$, ****; $p<0.0001$. N=5 per group.

Supplementary Figure 6

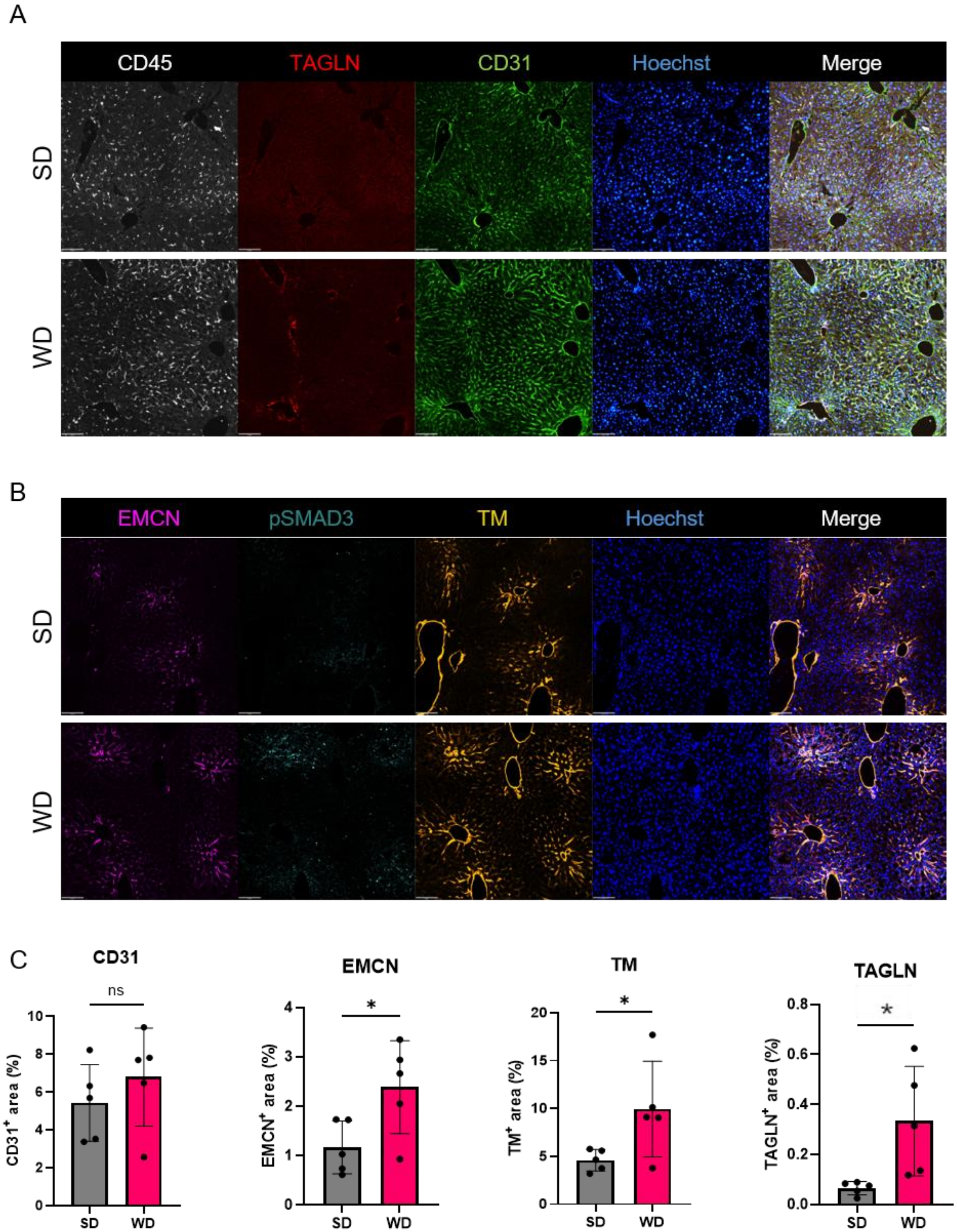

Supplementary Figure 6. Changes in vascular patterning in response to 12w WD. (A, B) Representative confocal IF images of liver sections from mice on a standard diet (SD) or Western diet (WD) for 12 weeks stained for (A) CD45 (white), transgelin (TAGLN) (red) and CD31 (green) and (B) endomucin (EMCN) (red), pSMAD3 (turquoise) and thrombomodulin (TM) (yellow). Scale bars= 200µm. (C) Quantification of positive fluorescence signal for CD31, EMCN, TM, and TAGLN. For statistical analysis, unpaired t-tests were used. ns; not significant, *; $p<0.05$. N=5 per group.

Supplementary Figure 7

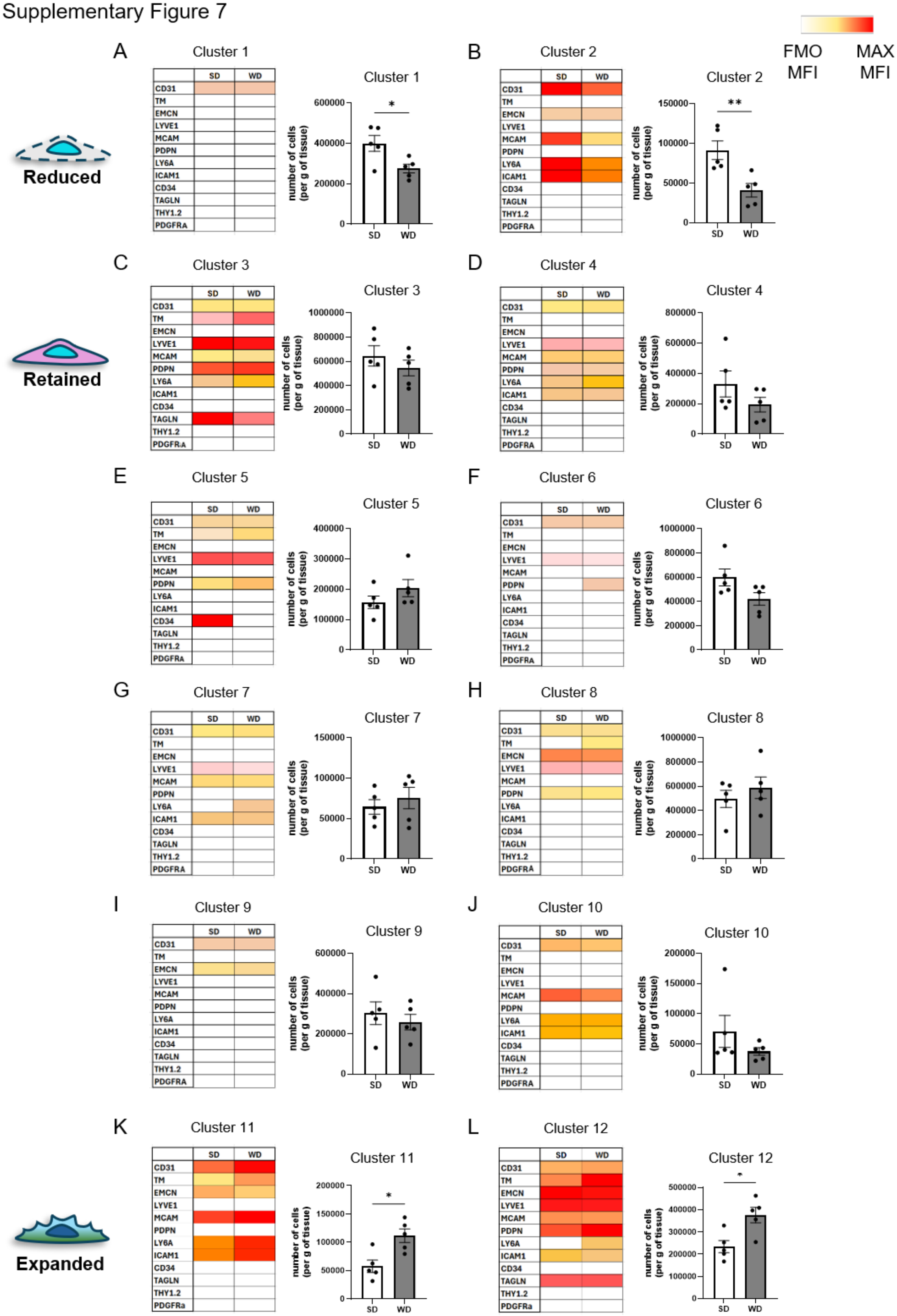

Supplementary Figure 7. Heatmaps of the phenotypic properties of each EC cluster split by diet group. Heatmaps of median fluorescence intensity (MFI) for animals on standard diet (SD) or Western diet (WD) for (A, B) Reduced clusters 1 and 2, (C-J) Retained clusters 3-10 and (K, L) Expanded clusters 11 and 12. The heatmaps were created using full spectrum flow cytometry data. The fluorescence minus one (FMO) MFI for each marker was used as the threshold for positive signal. *; $p<0.05$, **; $p<0.01$. N=5 per group.

Supplementary Figure 8

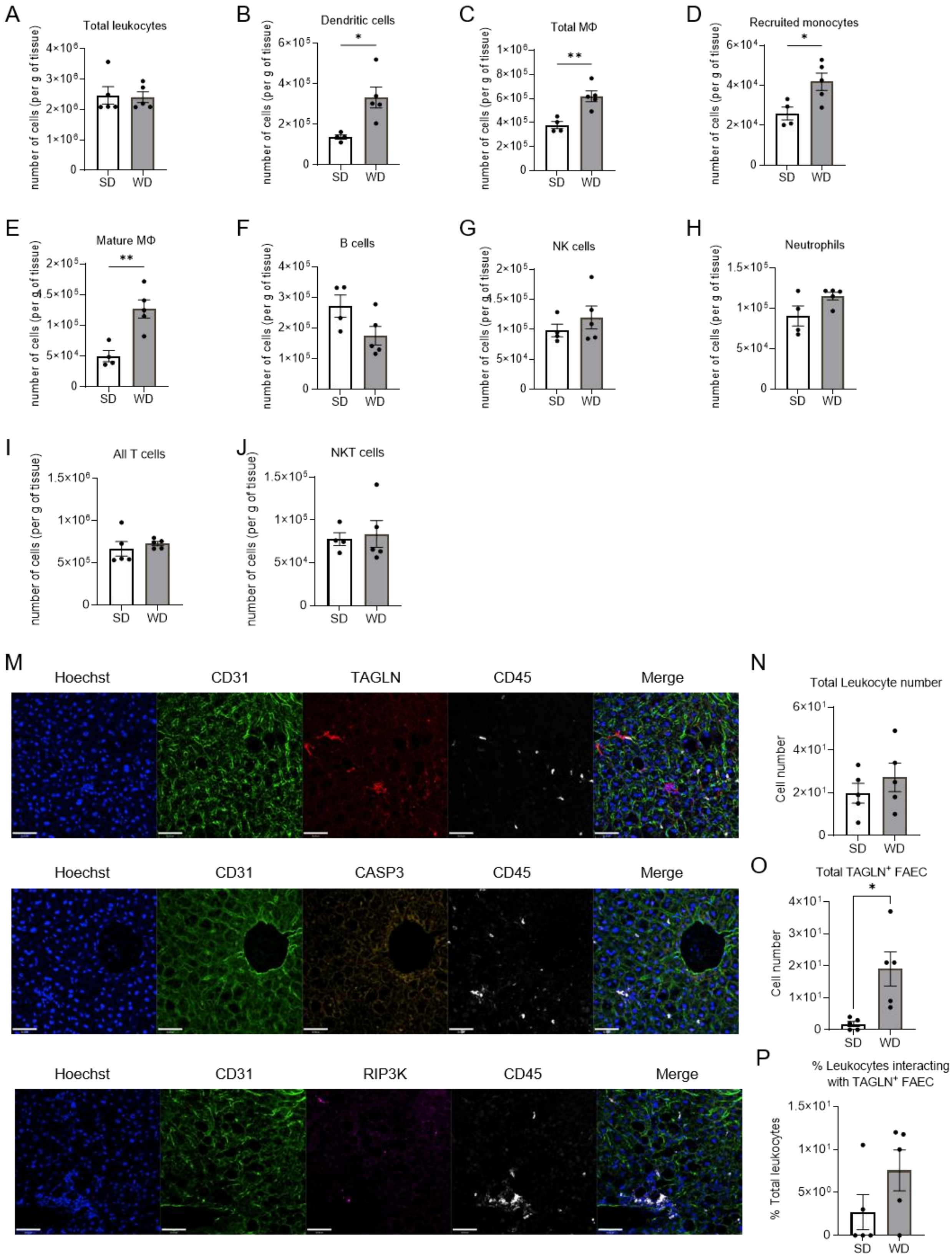

Supplementary Figure 8. Immune cell full spectrum flow cytometry profile and leukocyte interactions in response to 12w WD. (A-J) Quantification of (A) total number of leukocytes, (B) dendritic cells, (C) B cells, (D) NK cells, (E) macrophages, (F) recruited monocytes, (F) mature macrophages, (H) neutrophils, (I) All T cells and (J) NK T cells within the liver following standard diet (SD) or Western diet (WD). For statistical analysis, t-tests were used. (M) Top to bottom shows representative split images of TAGLN (red), CASP3 (orange) and RIPK3 (magenta) with nuclei (blue), CD31 (green) and CD45 (white). Quantification of total (N) $CD45^+$ and (O) $TAGLN^+$ FAEC objects counted per field for SD and 12 WD animals. (P) Quantification of the percentage total $CD45^+$ leukocytes which are touching $TAGLN^+$ FAEC. N=5 mice per group. For statistical analysis one-way ANOVA tests with Tukey's multiple comparisons test were used. N=5 animals/group. *; p<0.05, **; p<0.01.

Supplementary Figure 9

A

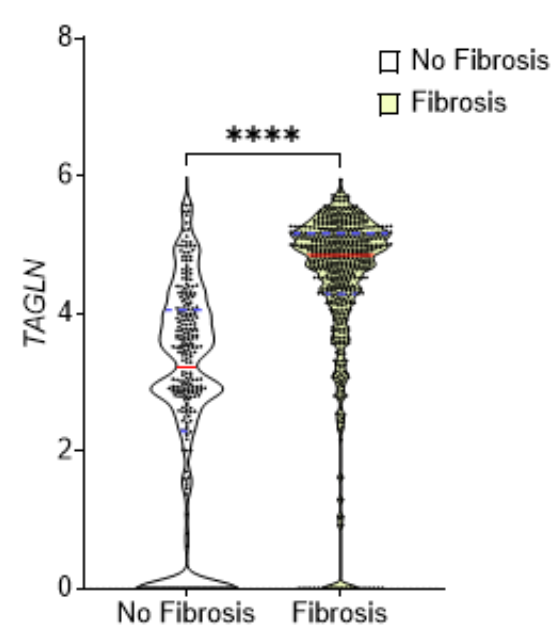

B

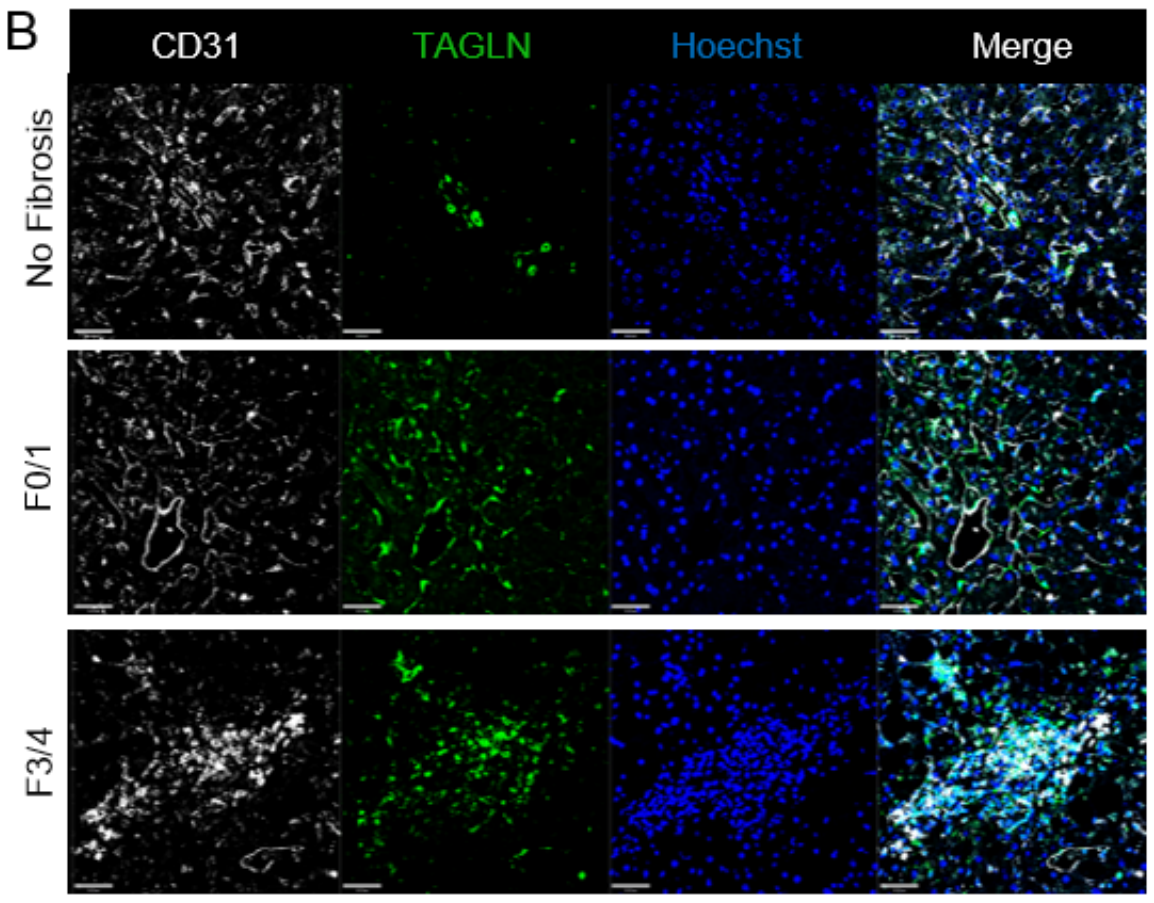

C

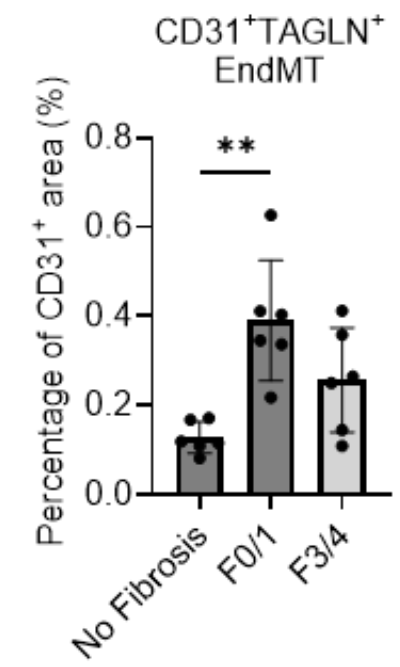

D

1606 DEG

EC Identity

Gene	avg_log2FC
MCAM	5.65
CDH5	2.17
PLVAP	2.08
VWF	2.08
ERG	2.05
EMCN	1.99
EGFL7	1.94
ENG	1.73
CD34	1.70
FLT1	1.43

EndMT

Gene	avg_log2FC
CNN1	9.32
ACTA2	9.13
TAGLN	7.80
TGFB1I1	5.27
PDGFA	5.27
THY1	3.88
COL6A2	2.48
FN1	1.62
VIM	1.41
SNAI1	0.98

Inflammation

Gene	avg_log2FC
ITGA7	6.10
ADAMTS12	5.44
ADAMTS5	3.92
LAMA5	3.64
LAMA3	3.54
ADAMTS1	2.76
ITGA3	2.72
HSPG2	2.42
CXCL12	2.16
THBS1	1.81

E

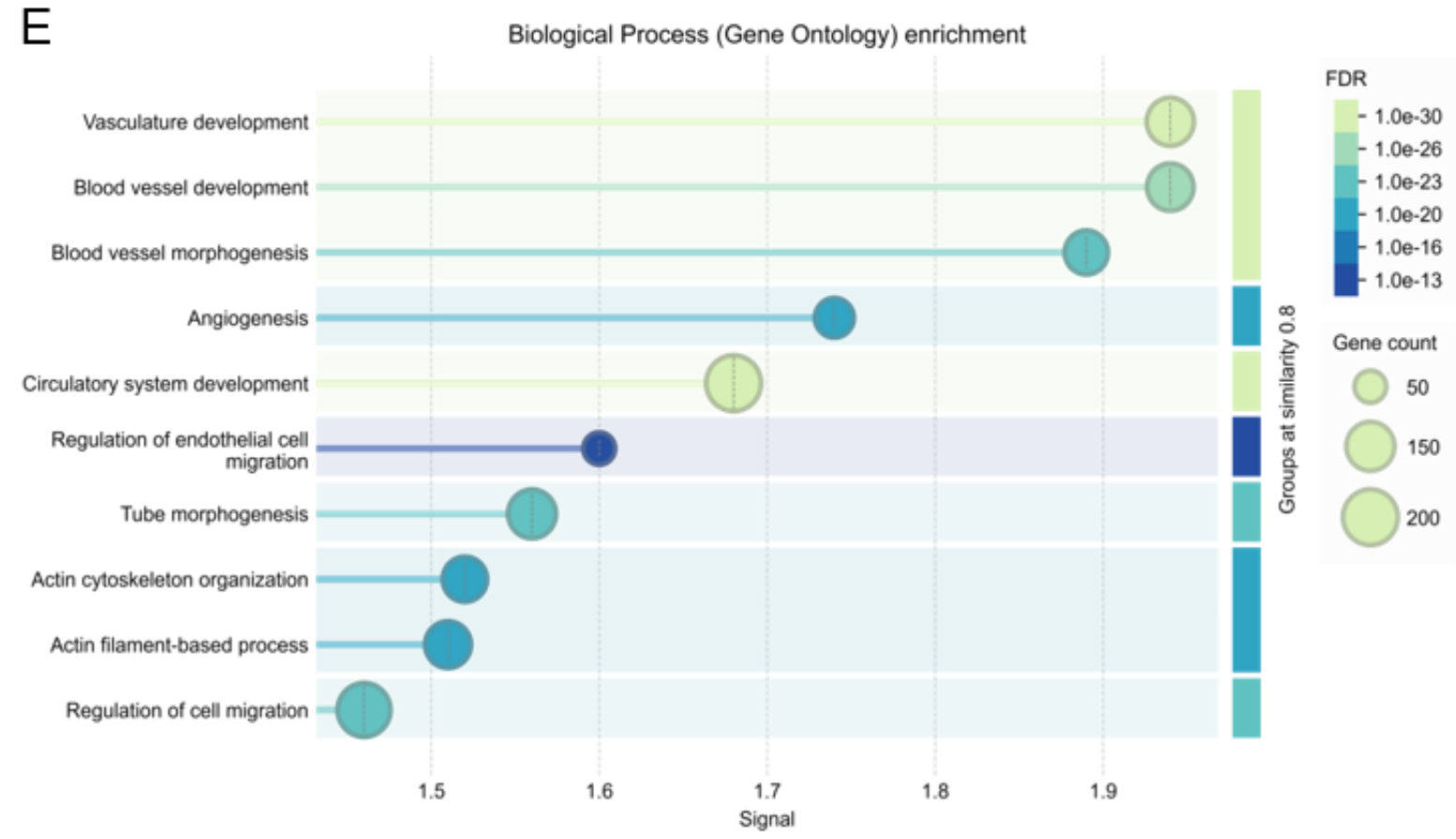

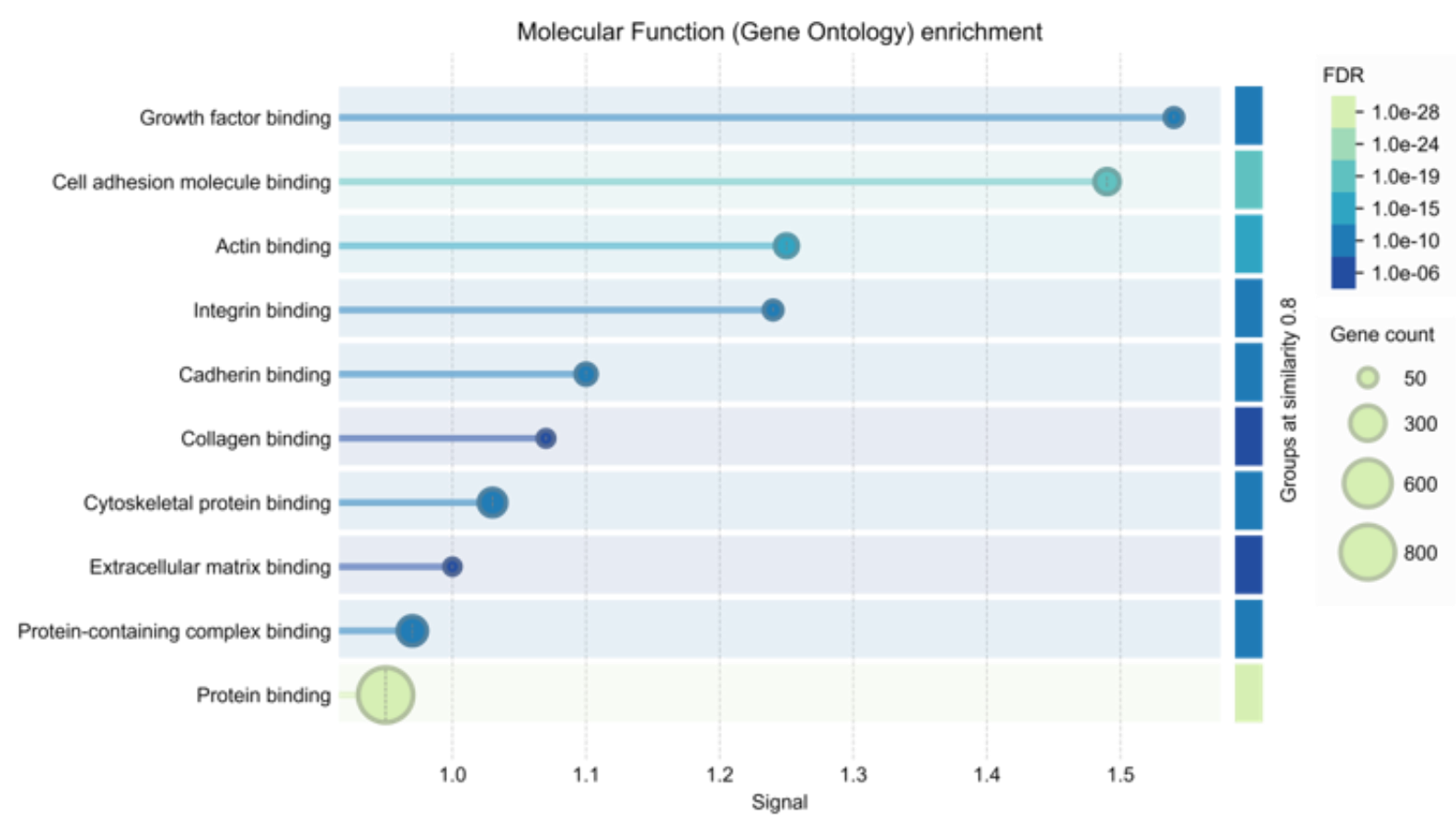

F

	Phenotypes		
**Nomenclature of Expanded FAEC subpopulations**	**CCl_4**	**Western Diet**	**Bariatric Surgery**
**THY1.2+ FAEC**	CD31+, THY1.2+ ICAM1+	x	x
**TAGLN+ FAEC**	CD31+, TAGLN+ MCAM+ LY6A+	CD31+, TAGLN+ MCAM+ LY6A+ ICAM1+	CD31+, TAGLN+ MCAM+

Supplementary Figure 9. Expression of TAGLN+ FAEC is a conserved feature of MASLD with the potential to be a biomarker of progressive disease. (A) Violin plot of scRNAseq analysis of cell number (dots) and median expression profile of $TAGLN^+$ EC from liver biopsies from MASLD patients after bariatric surgery. (B) Representative confocal images of patient biopsies stained for CD31 (white), transgelin (TAGLN) (green) and nuclei (Hoechst; blue). Scale bar= 50 µm. (C) Quantification of $CD31^+TAGLN^+$ EndMT cells expressed as a percentage of total $CD31^+$ area (N=6 patients per group). (D) Gene enrichment analysis of $TAGLN^+$ FAEC presented as a Table of top 10 enriched EC identity genes, EndMT genes and inflammation identified using scRNAseq. (E) Gene ontology analysis of all significantly enriched genes (Log2FC >0.5, P adj <0.05) for Biological Processes and Molecular Function. (F) Summary table phenotype of detailing the expanded FAEC subpopulations across the two pre-clinical models and bariatric surgery patient group. For statistical analysis (A) unpaired Welch's t-test or (C) one-way ANOVA tests with Tukey's multiple comparisons test were used. N=6 per patient stratification.

Supplementary Figure 10

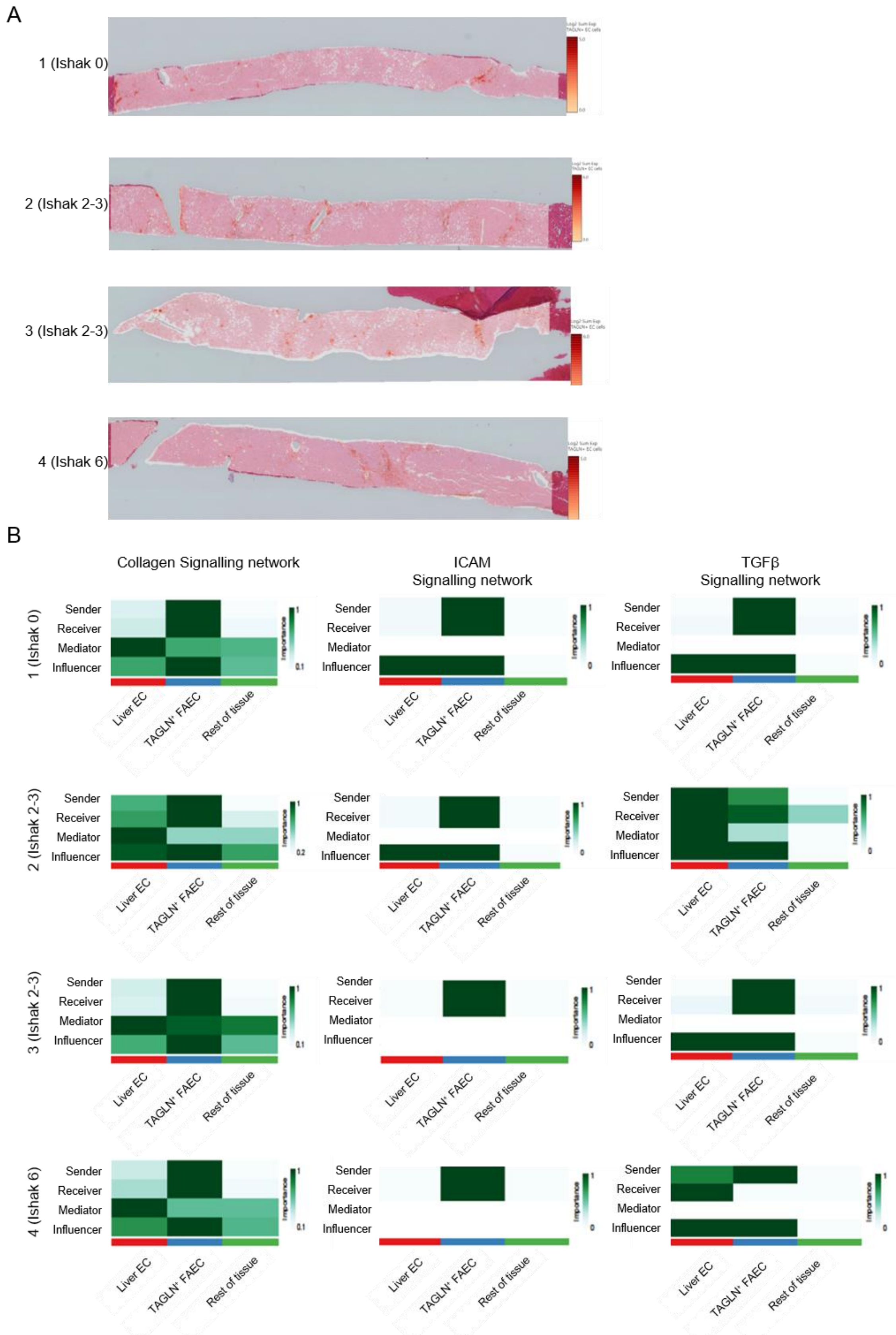

Supplementary Figure 10. Spatial Transcriptomic analysis of four MASLD patients. (A) Localisation of $TAGLN^{+}$ FAEC in patients with different stratification from F0-F4. (B) Heatmap of enriched Collagen, ICAM and TGFβ signalling pathways per patient.

Supplementary Figure 11

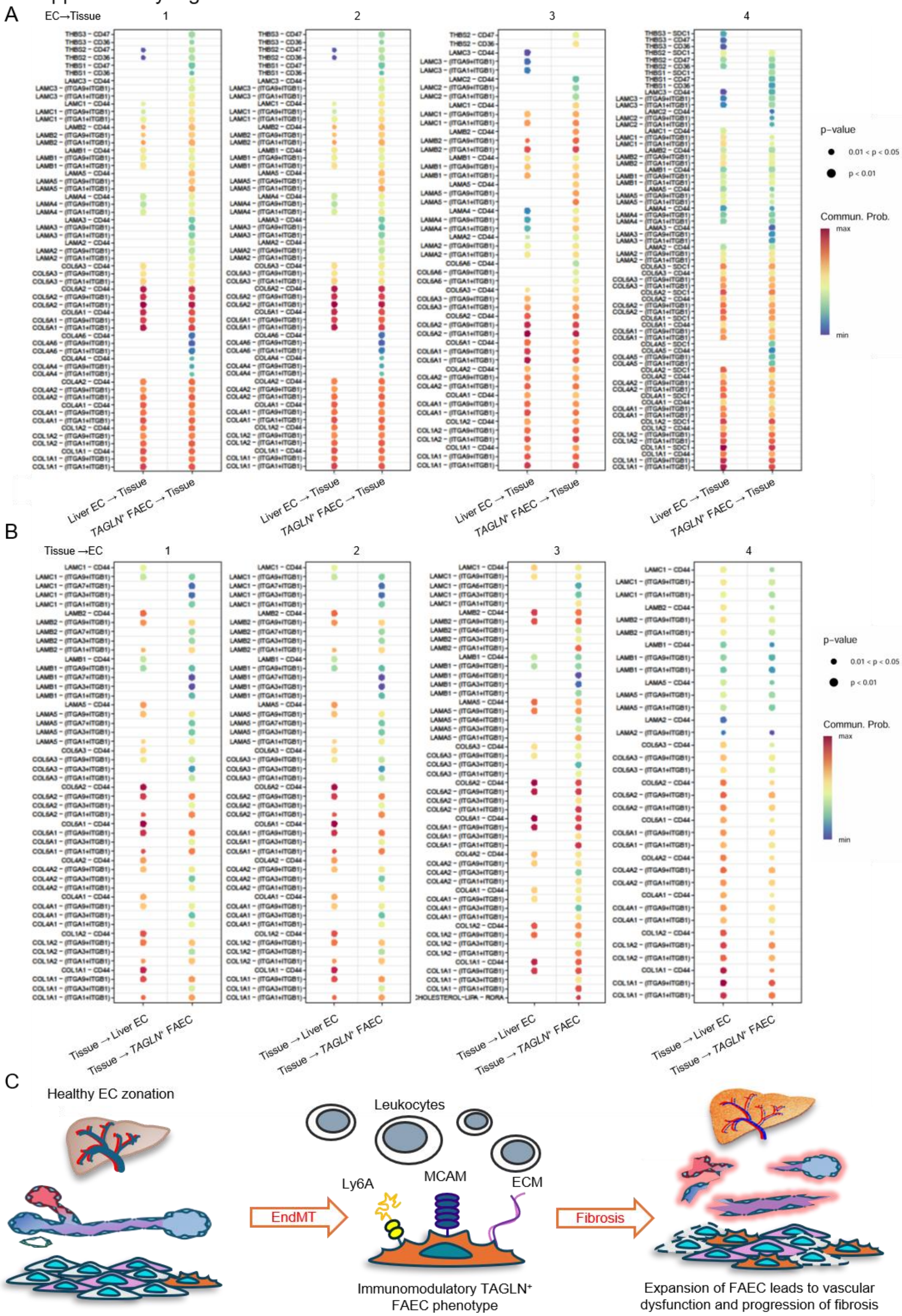

Supplementary Figure 11. Cell chat analysis of four MASLD patients. CellChat analysis for each patient for (A) Liver EC vs TAGLN$^+$ FAEC to tissue and (B) tissue to Liver EC vs TAGLN$^+$ FAEC. (C) Summary schematic of the role of FAEC in the development and progression of vascular dysfunction, fibrosis and MASLD.

Supplementary Table 1

Biopsy ID	Diagnosis	Age (years)	Ethnicity	Sex (F/M)	Ethanol (ml/ week)	BMI (kg/m^2)	Liver Stiffness Measurement (kPa)	Liver Steatosis Measurement (dB/m)	Histological fibrosis stage (Ishak)	FIB-4	Co-morbidities
1	MASLD	53	White-British	F	170	48.08	12.6	265	0	0.64	Hypertension, Type 2 Diabetes, Atrial Fibrillation, previously resected thyroid cancer
2	MASLD	56	White-British	M	0	44.26	33.0	382	2-3	1.28	Depression, Hypertension, Gout, Obstructive Sleep Apnoea, Osteoarthritis
3	MASLD	21	Mixed – White and Asian	F	0	22.30	16.5	258	2-3	0.58	Depression
4	MASLD	48	White-British	M	80	23.13	15.8	332	6	3.37	Nocturnal epilepsy

Supplementary Table 1. Patient profile for spatial transcriptomic data.

Supplementary Table 2

Target	Host	Clonality	Fluorophore	Dilution	Product number	Supplier
CD31	Goat	polyclonal	AF488	1:100	FAB3628G	R&D Systems
CD31	Sheep	polyclonal	unconjugated	1:100	AF806	R&D Systems
TM	Goat	polyclonal	unconjugated	1:500	AF3894	R&D Systems
EMCN	Rat	Monoclonal (V.7C7.1)	unconjugated	1:200	ab106100	Abcam
LYVE1	Rat	Monoclonal (ALY7)	unconjugated	1:100	48-0443-82	Invitrogen
MCAM	Rat	Monoclonal (ME-9F1)	unconjugated	1:100	130-101-866	Miltenyi
TAGLN	Rabbit	Polyclonal	unconjugated	1:500	ab14106	Abcam
THY1.2	Rat	Monoclonal (G7)	unconjugated	1:100	105201	Biolegend
phospho-SMAD3	Rabbit	Monoclonal (EP823Y)	unconjugated	1:100	ab52903	Abcam
CD45	Rat	F30-11	biotinylated	1:100	103104	Biolegend
CASP3	Rabbit	Polyclonal	unconjugated	1:100	9661	Cell Signalling
RIPK3	Rabbit	Monoclonal	unconjugated	1:100	91702	Cell Signalling
Albumin	Goat	Polyclonal	unconjugated	1in 100	NB600-41532	Novus

Host	Reactivity	Fluorophore	Dilution	Product number	Supplier
donkey	goat	488	1:1000	A32814	Invitrogen
donkey	goat	555	1:1000	A21432	Invitrogen
donkey	rabbit	488	1:1000	A21206	Invitrogen
donkey	rabbit	555	1:1000	A32794	Invitrogen
donkey	rabbit	647	1:1000	A31573	Invitrogen
donkey	rat	488	1:1000	A21208	Invitrogen
donkey	rat	550	1:1000	SA5-10027	Invitrogen
chicken	rat	647	1:1000	A21472	Invitrogen
streptavidin	biotin	555	1:1000	S32355	Invitrogen

Supplementary Table 2. List of primary (top) and secondary (bottom) antibodies used for tissue immunofluorescence experiments. (N/A: not applicable, AF: Alexa Fluor)

Supplementary Table 3

Target	Fluorophore	Clone	Dilution for liver samples	Dilution for heart samples	Product number	Supplier
CD31	BV785	390	1:320	1:640	102435	Biolegend
CD34	BV421	MEC14.7	1:160	1:80	119321	Biolegend
CD45	BUV395	30-F11	1:640	1:640	564279	BD Biosciences
EMCN	eFluor660	V7G7	1:640	1:320	50-5851-82	Invitrogen
ICAM1	PerCP-Cy5.5	YN1/1.7.4	1:320	1:160	116124	Biolegend
LY6A	BV480	D7	1:320	1:640	746663	BD Biosciences
LYVE1	PE-Cy5.5	ALY7	1:640	1:640	NBP1-43411	Novus Biologics
MCAM	BV650	ME-9F1	1:320	1:640	740636	BD Biosciences
PDGFRA	APC-eFluor780	APA7	1:160	1:640	47-1401-82	Thermo Scientific
PDPN	PE-Cy7	8.1.1	1:320	1:640	127411	Biolegend
TAGLN	AF488	TAGLN/247	1:320	1:320	NBP2-47688	Novus Bio
THY1.2	AF700	30-H12	1:640	1:640	105320	Biolegend
TM	PE	461714	1:160	1:640	FAB3894P	Biotechne

Supplementary Table 3. Antibody details for the endothelial panel used for full spectrum flow cytometry.

Supplementary Table 4

Target	Fluorophore	Clone	Dilution for liver samples	Product number	Supplier
B220	Qdot655	RA3-6B2	1:320	Q10176	Thermo Scientific
CD11b	APC-Fire810	M1/70	1:640	101288	Biolegend
CD11c	BUV563	N418	1:320	749040	BD Biosciences
CD19	Pacific Blue	6D5	1:80	115523	Biolegend
CD3	Alexa Fluor 647	17A2	1:640	100209	Biolegend
CD31	PE-Cy7	390	1:160	25-0311-81	eBioscience
CD4	PE	RM4-5	1:320	12-0042-82	Thermo Scientific
CD45	BUV395	30-F11	1:640	564279	BD Biosciences
CD8a	BV421	53-6.7	1:160	100737	Biolegend
CX3CR1	PerCP-Cy5.5	SA011F11	1:80	149009	Biolegend
F4/80	PE-CF594	T45-2342	1:320	565613	BD Biosciences
Ly6A	BV480	D7	1:640	746663	Biolegend
Ly6C	BV605	HK1.4	1:320	128035	Biolegend
Ly6G	FITC	1A8	1:640	127605	Biolegend
MHC II	Alexa Fluor 700	M5/114.1.5.2	1:640	56-5321-82	Thermo Scientific
CD64	BV510	X54-5/7.1	1:160	139335	Biolegend
TCRβ	BV570	H57-597	1:80	109231	Biolegend
XCR1	SparkUV 387	ZET	1:160	148235	Biolegend
TIM4	APC	RMT4-54	1:640	130021	Biolegend
NK1.1	BV785	V15	1:160	108749	Biolegend

Supplementary Table 4. Antibody details for the immune cell panel used for full spectrum flow cytometry.
